# Identification and characterization of hippuristanol-resistant mutants reveals eIF4A1 dependencies within mRNA 5′ leader regions

**DOI:** 10.1093/nar/gkaa662

**Published:** 2020-08-07

**Authors:** Jutta Steinberger, Leo Shen, Stephen J. Kiniry, Sai Kiran Naineni, Regina Cencic, Mehdi Amiri, Sarah A E Aboushawareb, Jennifer Chu, Rayelle Itoua Maïga, Brahm J Yachnin, Francis Robert, Nahum Sonenberg, Pavel V Baranov, Jerry Pelletier

**Affiliations:** Department of Biochemistry, McGill University, Montreal H3G 1Y6, Canada; Department of Biochemistry, McGill University, Montreal H3G 1Y6, Canada; School of Biochemistry and Cell Biology, University College Cork, Cork, Ireland; Department of Biochemistry, McGill University, Montreal H3G 1Y6, Canada; Department of Biochemistry, McGill University, Montreal H3G 1Y6, Canada; Department of Biochemistry, McGill University, Montreal H3G 1Y6, Canada; Department of Biochemistry, McGill University, Montreal H3G 1Y6, Canada; Department of Biochemistry, McGill University, Montreal H3G 1Y6, Canada; Department of Biochemistry, McGill University, Montreal H3G 1Y6, Canada; Department of Chemistry & Chemical Biology & the Institute for Quantitative Biomedicine, Rutgers The State University of New Jersey, Piscataway 08854, NJ; Department of Biochemistry, McGill University, Montreal H3G 1Y6, Canada; Department of Biochemistry, McGill University, Montreal H3G 1Y6, Canada; Rosalind and Morris Goodman Cancer Research Center, McGill University, Montreal H3A 1A3, Canada; School of Biochemistry and Cell Biology, University College Cork, Cork, Ireland; Shemyakin-Ovchinnikov Institute of Bioorganic Chemistry RAS, Moscow, Russia; Department of Biochemistry, McGill University, Montreal H3G 1Y6, Canada; Rosalind and Morris Goodman Cancer Research Center, McGill University, Montreal H3A 1A3, Canada; Department of Oncology, McGill University, Montreal H3G 1Y6, Canada

## Abstract

Hippuristanol (Hipp) is a natural product that selectively inhibits protein synthesis by targeting eukaryotic initiation factor (eIF) 4A, a DEAD-box RNA helicase required for ribosome recruitment to mRNA templates. Hipp binds to the carboxyl-terminal domain of eIF4A, locks it in a closed conformation, and inhibits its RNA binding. The dependencies of mRNAs for eIF4A during initiation is contingent on the degree of secondary structure within their 5′ leader region. Interest in targeting eIF4A therapeutically in cancer and viral-infected settings stems from the dependencies that certain cellular (e.g. pro-oncogenic, pro-survival) and viral mRNAs show towards eIF4A. Using a CRISPR/Cas9-based variomics screen, we identify functional *EIF4A1* Hipp-resistant alleles, which in turn allowed us to link the translation-inhibitory and cytotoxic properties of Hipp to eIF4A1 target engagement. Genome-wide translational profiling in the absence or presence of Hipp were undertaken and our validation studies provided insight into the structure-activity relationships of eIF4A-dependent mRNAs. We find that mRNA 5′ leader length, overall secondary structure and cytosine content are defining features of Hipp-dependent mRNAs.

## INTRODUCTION

Demonstrating that the bioactivity of a small molecule is mediated through a specific target or set of targets, is a crucial step in the drug discovery and development process but is arduous and time-consuming. Incomplete or incorrect target information of a small molecule can be quite costly and can lead to failures in clinical trials. Yeast genetics, libraries of cDNA variant alleles, and CRISPR/Cas9 mutagenesis are but some of the approaches that can be employed to link compound activity to a specific biological target (1–3). When genetic data is combined with biochemical characterization of compound-target interactions, it can provide a more holistic view that is essential to guide future developments.

During the course of studies aimed at identifying cancer cell vulnerabilities, we and others have identified an opportunity to decrease cancer cell survival and perturb homeostasis by inhibiting a central regulatory node of translation governed by eukaryotic initiation factor (eIF) 4F (4). This key factor catalyzes the recruitment of ribosomes to capped messenger RNAs (mRNAs) and is comprised of eIF4E (a cap-binding protein), eIF4A (a DEAD-box RNA helicase) and eIF4G (a larger scaffolding protein with RNA-binding activity). Mammalian cells encode two eIF4A paralogs, eIF4A1 [DDX2A] and eIF4A2 [DDX2B], which share 90% identity and can both associate with the eIF4F complex (4). eIF4A1 is generally the more abundant protein and is more intensively studied since, unlike eIF4A2, it is essential for cell viability (5). The role of eIF4A in the initiation process is not completely understood, but the dependency of mRNAs with elevated secondary structure within their 5′ leader region on eIF4A activity (6) implicate it in the unwinding of mRNA templates for ribosome recruitment and binding (6). The degree to which an mRNA is dependent on eIF4F for ribosome recruitment is thought to be a key determinant in dictating translational efficiency and offers an opportunity to influence mRNA translational output in a rheostatic manner through eIF4F activity.

We and others have identified several compounds that target different activities of eIF4F, including the occlusion of eIF4E cap-binding (7), disruption of eIF4E:eIF4G association (8,9), reduction of eIF4E mRNA levels using anti-sense oligonucleotides (10), and inhibition of eIF4A helicase activity (11–14). Among these inhibitors are three classes of compounds that target eIF4A: hippuristanol (Hipp) (12), rocaglates (13,15), and pateamine A (and analogs) (11,14). These compounds have very different modes of action. Rocaglates and pateamine A cause clamping of eIF4A and eIF4F to RNA and interfere in a dominant manner with ribosome recruitment (11,14,16–18). On the other hand, Hipp binds to the eIF4A1 and eIF4A2 carboxy-terminal domain (CTD) and inhibits RNA binding, thus blocking the ribosome recruitment process (12).

**Figure 1.**
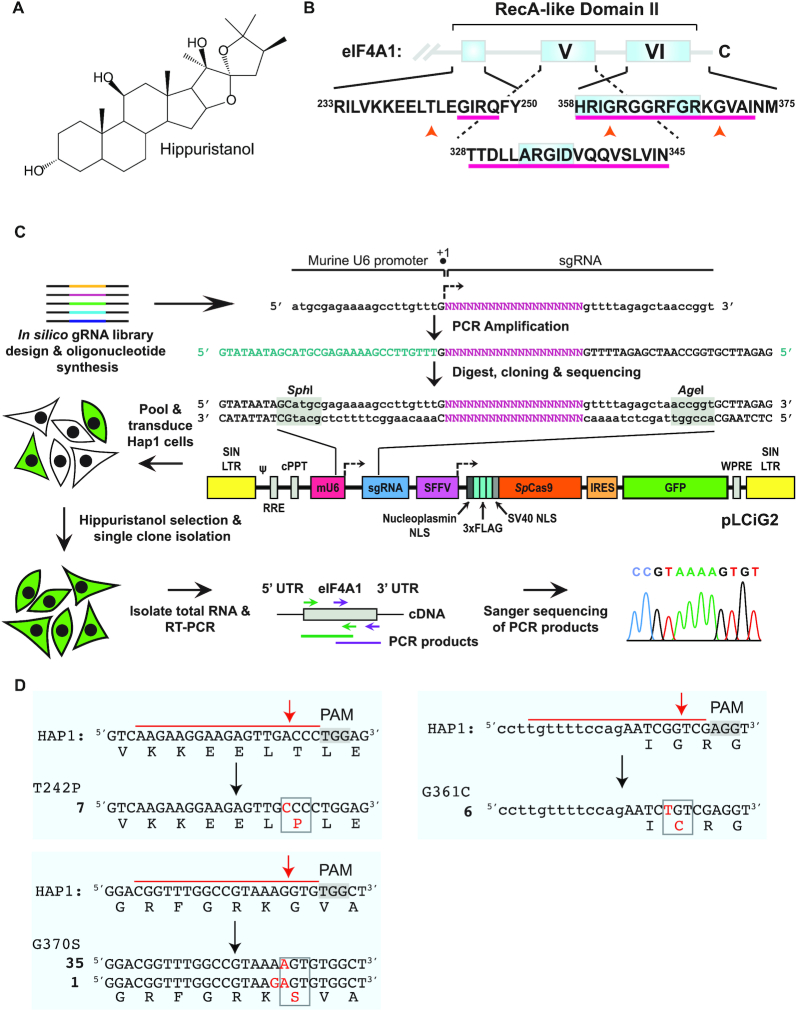
Generation and characterization of Hipp^R^ variants in Hap1 cells using CRISPR/Cas9- mediated NHEJ. (**A**) Chemical structure of hippuristanol. (**B**) Schematic diagram showing location of the Hipp-binding site within the eIF4A1 (and eIF4A2) CTD (denoted by a magenta underline). The location of the three amino acid substitutions found in this study to confer Hipp-resistance are indicated by orange upward arrowheads. The relative position of conserved motifs V and VI are provided for reference and are boxed in grey (57). (**C**) Strategy undertaken to generate an SpCas9 sgRNA library targeting eIF4A1 coding exons. sgRNAs predicted to target *EIF4A1* (NM_001416.3) by CHOPCHOP (https://chopchop.cbu.uib.no/) were shotgun-cloned into pLCiG2 using unique *Sph*I and *Age*I sites. Following sequence verification, the pooled library was used to generate lentivirus which in turn was used to infect Hap1 cells. Cells were selected in 300 nM Hipp, after which single colonies were picked and expanded. The *EIF4A1* cDNA was cloned from all cell lines and characterized by Sanger sequencing. (**D**) The set of *EIF4A1* missense mutations identified in Hipp^R^ Hap1 cells. The target PAM is shaded, the nucleotides spanned by the sgRNA are denoted by a red overline, the nucleotide and amino acid substitutions are indicated in red and boxed in grey. Upper-case indicates exon-encoded nucleotides whereas lower-case is intron-derived sequences. The bold number to the left of the mutant sequence indicates the number of independently identified Hap1 clones harboring that specific mutation.

Hipp (Figure 1A) is a natural product initially isolated from *Isis hippuris* (19), a bamboo coral that abolishes eIF4A’s RNA-binding activity by locking the helicase in a closed conformation (20). Through structural studies, Hipp was shown to interact with amino acids that are conserved from yeast to mammals within, or adjacent to, motifs present in the eIF4A1 and eIF4A2 CTD (Figure 1B). This binding site is not conserved among other DEAD-box RNA helicases, thus providing a rationale for Hipp's selectivity (21). Despite extensive studies examining Hipp's effects on eIF4A, it still remains an open question as to whether its cytotoxicity towards cells is a consequence of eIF4A inhibition. In this study, we describe the implementation of a CRISPR/Cas9 variomics screen (3) to isolate Hipp-resistant (Hipp^R^) alleles of *EIF4A1* that maintain all cellular activities required for wild-type function. Subsequent biochemical characterization of these variants allowed us to determine that Hipp bioactivity is intimately linked to inhibition of eIF4A1 activity. Through a transcriptome- and translatome-wide analysis, we identified Hipp-responsive mRNAs in Hap1 chronic myelogenous leukemia cells and define 5′ leader length, overall leader secondary structure, and cytosine content as characteristics of Hipp-responsive mRNAs.

## MATERIALS AND METHODS

### Hippuristanol

The complete synthesis of hippuristanol has been previously described (22). Stocks were prepared in 100% DMSO, aliquoted, and stored at −80°C.

### Cell culture, sgRNA library generation and screening parameters

Hap1 cells were cultured in IMDM supplemented with 10% fetal bovine serum (FBS), 100 U/mL penicillin/streptomycin and 2 mM l-glutamine at 37°C and 5% CO_2_. HEK-293/17 and NIH/3T3 cells were grown in DMEM supplemented with 10% FBS, 100 U/ml penicillin/streptomycin and 2 mM l-glutamine at 37°C and 5% CO_2_.

Target sites for CRISPR/SpCas9 and Cpf1-directed mutagenesis were selected using the CHOPCHOP online tool (https://chopchop.cbu.uib.no). Oligos for generation of the sgRNA libraries were ordered individually from BioCorp, amplified using PCR primers for Cas9 library: Fwd 5′-GTATAATAGCATGCGAGAAAAGCCTTGTTT-3′ and Rev 5′-CTCTAAGCACCGGTTAGCTCTAAAAC-3′; primers for Cpf1 library: Fwd 5′GTATAATAGCATGCAGAAAAGCCTTGTTTG-3′ and Rev 5′CTCTAAGCGTATCGGCCACTCGAG-3′, and cloned into the pLCiG2 backbone via *Sph*I and *Age*I restriction sites for the Cas9 library (see Supplementary Table S1) or into the pLmU6/Cpf/iG2 backbone via *Sph*I and *Xho*I restriction sites for the Cpf1 library (see Supplementary Table S2). Clones were individually isolated, sequence-verified and arrayed. For library screening, individual clones were grown overnight in 96-well deep well plates, pooled, and grown for an additional 4 h, at which point the plasmid DNA was isolated. The resulting library was then used to prepare virus. Transduced Hap1 cells were exposed to 300 nM Hipp for 10 days and the Hipp-resistant colonies that emerged were individually picked. Mutations in the *EIF4A1* gene were assessed by Sanger sequencing at the gDNA and RNA level.

### Genomic DNA isolation

Cells were collected by centrifugation at 300 × g for 10 min, washed in PBS, and resuspended in 500 μl DNA extraction buffer (0.2% SDS, 5 mM EDTA, 200 mM NaCl, 100 mM Tris–HCl [pH8.5], 50 μg Proteinase K). Lysates were incubated at 55°C overnight and the DNA was precipitated by adding one volume of isopropanol. After washing with 70% ethanol, the pellet was resuspended in TE (10 mM Tris [pH 8.0], 1 mM EDTA).

### Sulforhodamine B (SRB) assay

Cells (10 000/well) were seeded in a 96-well plate and exposed to various concentrations of compound or vehicle (DMSO) for 48 h. Subsequently, cells were washed with PBS, fixed with 50% TCA for 45 min and stained with 0.4% SRB for at least 15 min. Plates were washed four times with 1% acetic acid then dried. The remaining dye was resuspended in 100 μl 10 mM Tris [pH 9] per well. The absorbance at 550 nm (OD_550_) was measured using a microplate reader (SpectraMax M5, Molecular Devices) and the relative viability was calculated by normalizing to the DMSO controls.

### Colony formation assay

Twenty thousand cells/well in six-well plates were seeded one day prior to starting treatment. Cells were treated with vehicle or 100 nM hippuristanol for 6 days and medium refreshed once after 3 days. At the end of treatment, cells were fixed with 4% formaldehyde for >1 h at RT. Fixed cells were then stained with 4% crystal violet overnight at RT. After staining, plates were rinsed with tap water and air dried.

### Cellular thermal shift assay (CETSA)

Cells from a 15-cm dish were trypsinized and counted. Cells were washed in 10 ml Hank's Salt Solution and the pellet resuspended in 1/10th volume of Hank's Salt Solution. Approximately 2 × 10^6^ cells (95 μl) were transferred to each well of a 96-well PCR plate containing 5 μl of pre-aliquoted compound. Cells were incubated at 37°C for 30 min using a PCR machine (Mastercycler Pro, Eppendorf), cooled to 22°C over 3 min and incubated another 3 min at 22°C. A multi-channel pipette was used to transfer 25 μl of 5× protease inhibitor mix to each well. The plate was resealed and cells were lysed by freeze-thawing three times in a dry ice/methanol bath for 1 min followed by incubation at 37°C for 1.5 min. Lysates were then transferred to Eppendorf tubes and centrifuged at 14 000 g for 20 min at 4°C. The supernatant (100 μl) was transferred to a new Eppendorf tube containing 50 μl of pre-aliquoted 3× SDS sample buffer. Samples were heated for 5 min at 95°C and used for SDS-PAGE analyses.

### Differential scanning fluorimetry (DSF)

Experiments were performed by incubating 2 μM of recombinant eIF4A1 (wild type, T242P, G361C or G370S) with 10 μM Hipp in DSF buffer (20 mM HEPES–KOH [pH 7.5], 70 mM KCl, 2 mM DTT, 1 mM Mg(OAc)_2_, 1 mM ATP, 7.5× Sypro Orange (S-6650, Thermo Fisher). Measurements were performed from 25°C to 70°C at a 1°C/min ramp rate using the CFX96 Touch™ Real-Time PCR Detection System (Bio-Rad). Data analysis was performed as described (23).

### Ribosome footprinting

Library preparation and data analysis were performed according to the method previously described (24) with the following modifications. Hap1 cells (12 × 10^6^) were seeded into 15-cm dishes the day before ribosome footprinting. Cells were treated with 50 nM Hipp for 1 h before harvesting. Plates were then placed on ice and washed twice with 15 ml cold PBS supplemented with 100 μg/ml cycloheximide (CHX). Cells were scraped in 250 μl of 3× Lysis buffer (1× Lysis buffer: 20 mM Tris [pH 7.4], 150 mM NaCl, 5 mM MgCl_2_, 100 μg/ml CHX, 1 mM DTT, 1× cOmplete™ Protease Inhibitor Cocktail) and transferred to Eppendorf tubes. Triton X-100 was added to a final concentration of 1% and vortexed briefly. The solution was centrifuged at 4°C for 2 min at 14 000 × g in a table-top centrifuge. 300 μg of sample was brought up to a volume of 500 μl and supplemented with 5.6 mM CaCl_2_. The sample was digested with 150 U Micrococcal nuclease (MNase) for 45 min at room temperate (RT), rotating end-over-end. The reaction was terminated by adding EGTA (pH 8) to a final concentration of 8.4 mM and incubated for 5 min at RT. Samples were loaded on 10–50% sucrose gradients, which were centrifuged at 36 000 rpm for 3 h using an SW40 rotor. Fractionated samples were frozen on dry ice immediately.

RNA from the monosome fractions, as well as from the cytoplasmic lysate, was isolated using TRIzol™ according to the manufacturer's instructions (Thermo Fisher Scientific). Ribo-seq library preparation was performed using 15 μg of RNA sample as described by Glincy and Ingolia (24). The RNA-seq library was prepared using the same protocol as Ribo-seq libraries with the following modifications. Ribosomal RNA was depleted using the NEB Next rRNA depletion kit from 1 μg total RNA and randomly fragmented using the NEBNext^®^ Magnesium RNA Fragmentation Module and purified by isopropanol precipitation.

### Bioinformatics analysis

Demultiplexing and adaptor sequence removal (5′-AGATCGGAAGAGCACACGTCTGAA-3′) was performed using Cutadapt (25). Ribosomal RNA was removed using bowtie (26). PCR duplicates were removed using UMI tools (27). The remaining reads were aligned to the Gencode version 32 transcriptome (28). A single representative transcript was chosen for each gene locus by selecting the principal isoform from the APPRIS database (29). For genes with multiple principal isoforms, a single transcript was selected based on the highest RNA-Seq TPM (transcripts per million). Differential expression/translation analysis was carried out using DESeq2 (30). Differences in the number of aligned reads with an adjusted *P*-value >0.05 were classified as non-significant, differences with an adjusted *P*-value <0.05 were classified as significant, and corresponding genes were classed as up-/down-regulated depending on whether the numbers of mapped reads were increased or decreased. The accession number for data originating from this study is GEO: GSE151687.

Alignments and differential gene expression analysis have been carried out as described above for the data from the following studies: Hsieh12 (GSE35469), Iwasaki16 (GSE70211), Rubio: (GSE61375) and Wolfe: (GSE56887) (Supplementary Figure S6). Transcripts with no mapped reads in one or more conditions were discarded. Spearman correlation between fold changes in the number of aligned reads for each individual transcript was used as a measure of differential gene expression similarity. G4Hunter (31) was used to find the number of G-quadruplexes per 1000 nts in both Hipp sensitive genes and 1000 equally sized groups of randomly selected transcripts. A window size of 25 and a threshold of 1.4 were used for both groups. Using the mean and standard deviation of the randomly selected groups, a *z*-score of 2.14 was calculated for the Hipp-sensitive genes.

MFE was calculated using the RNA fold function from the ViennaRNA python package (32). MFE values were corrected according to (33). For sliding windows, a window size of 20 nts with a step size of 2 nts was used.

### Cloning of Hipp-responsive 5′ leaders into bicistronic reporter

The 5′ leader sequences of the top seven Hipp-responsive transcripts (*WNK1, 4EBP2, PCBP2, ODC1, CCNG1, CCNI, CREBBP*) along with three negative controls (*ATP5PO, NDUFS6, 4EBP1*) (sequences in Supplementary Table S4) were designed with upstream *Nde*I and downstream *Mlu*I restriction sites and ordered from GenScript and BioCorp. The fragments were sub-cloned into pKS-FF-HCV-Ren bicistronic vectors. The same was done for the sequences used to investigate the effects of length and nucleotide composition. In brief, constructs of either 24 or 148 nts were designed using a random sequence generator that accepted user-input nucleotide compositions (https://users-birc.au.dk/∼palle/php/fabox/random_sequence_generator.php). Three sequences, specifically without ATG start codons, from each group were taken and cloned as described above. All sequences are in Supplementary Table S5.

### *In vitro* transcription

Reporters were linearized with *BamH*I, purified using the EZ-10 spin column DNA Cleanup Miniprep Kit (BioBasic, BS367) and 3 μg was transcribed with T3 RNA polymerase (NEB, M0378), 10× RNA polymerase buffer (NEB), 0.5 mM CTP, 0.5 mM ATP, 0.5 mM UTP, 0.1 mM GTP, 0.5 mM 3′-*O*-Me-^m7^GpppG (Anti-Reverse Cap Analog [ARCA]; NEB, S1411), 100 U RNase Inhibitor (NEB, M0307) in a volume of 100 μl for 3 h at 37°C. DNaseI (5U, NEB, M0303) was then added, and samples were incubated for an additional 30 min at 37°C. Standard phenol/chloroform extraction was performed. Samples were then purified through G-50 Sephadex columns followed by precipitation with ethanol and NH_4_OAc, and re-suspended in water. RNA was then quantified using a Nanodrop and stored at −80°C.

### *In cellula* translation experiments

HEK-293T/17 cells (2 × 10^5^) per well were seeded in 24-well plates the day before transfection. RNA (200 ng) was then transfected the next day using the DMRIE-C reagent (Invitrogen, 10459014) and Opti-MEM according to the manufacturer's instructions. Hippuristanol or DMSO (vehicle) was added to the transfected cells at the indicated concentration and in a final concentration of 0.25% DMSO. Cells were then harvested 7 h later using 40 μl Passive Lysis Buffer (Promega, E1941) and incubated at RT, shaking for 15 min. Lysates (20 μl) were then read using 100 μl of Firefly/Renilla substrates on a luminometer (Berthold Technologies Lumat LB 9507) (34).

## RESULTS

### A CRISPR/Cas9 variomics screen identifies *EIF4A1* Hipp^R^ alleles

To obtain *EIF4A1* Hipp^R^ alleles that are not compromised in biological activity, we undertook a variomics genetic screen. Our approach took advantage of the fact that during repair of double-stranded DNA breaks induced by Cas9, deletions/substitutions that maintain the reading frame while generating missense or in-frame mutations, occasionally arise (Supplementary Figure S1a) (3). To obtain estimates of the frequency of repair events that lead to substitution mutations, we designed a single guide RNA (sgRNA) for *S. pyogenes* (Sp) Cas9 (red) and Cpf1 (blue) targeting the eGFP open-reading frame (ORF) of a previously described traffic light reporter (TLR) (Supplementary Figure S1b) (35). Following Cas9- or Cpf1-mediated mutagenesis, we assessed the nature of the repair events directly by Sanger sequencing of PCR products obtained from the targeted region (Supplementary Figure S1b). The results indicate that base substitutions leading to missense and nonsense mutations comprise ∼2–5% of all repair products (Supplementary Figure S1c).

We then designed sgRNA libraries for SpCas9 and Cpf1 targeting all 11 human *EIF4A1* coding exons (Supplementary Tables S1 and S2). In total, 211 sgRNAs for SpCas9 and 41 sgRNAs for Cpf1 were generated and introduced into an All-in-One editing vector (36) (Figure 1C and Supplementary Figure S2). We chose to pursue the genetic screen in Hap1 cells, because of their near-haploid status (only a small duplication of chromosome 15 remains). Given the essential nature of *EIF4A1* (37) (located at 17p13), the screen design required the generation of Hipp^R^ alleles that can participate in all essential facets of eIF4A1 biology, otherwise no clones were expected to be recovered. Infections were performed to achieve a minimum representation of 10 ,000 cells/sgRNA during the screen and cells were placed under selection of 300 nM Hipp. Forty-nine colonies emerged from the SpCas9 library and none from the Cpf1 library nor from a parallel experiment where uninfected Hap1 cells were cultured in the presence of Hipp. Clones were characterized by sequencing RT-PCR products spanning the entire eIF4A1 ORF, followed by targeted PCR analysis of exons from genomic DNA (Figure 1C).

We identified three separate genetic lesions from this screen (*EIF4A1*^T242P^, *EIF4A1*^G361C^ and *EIF4A1*^G370S^) (Figure 1D). All mutation sites were targeted by an sgRNA present in the library, and the location of the modification occurred at the expected Cas9 cleavage site (Figure 1D). These mutations also map within, or proximally to, the previously defined CTD Hipp-binding site (Figure 1B, upward orange arrowheads).

### *In vitro* assessment of *EIF4A1* Hipp^R^ variants

**Figure 2.**
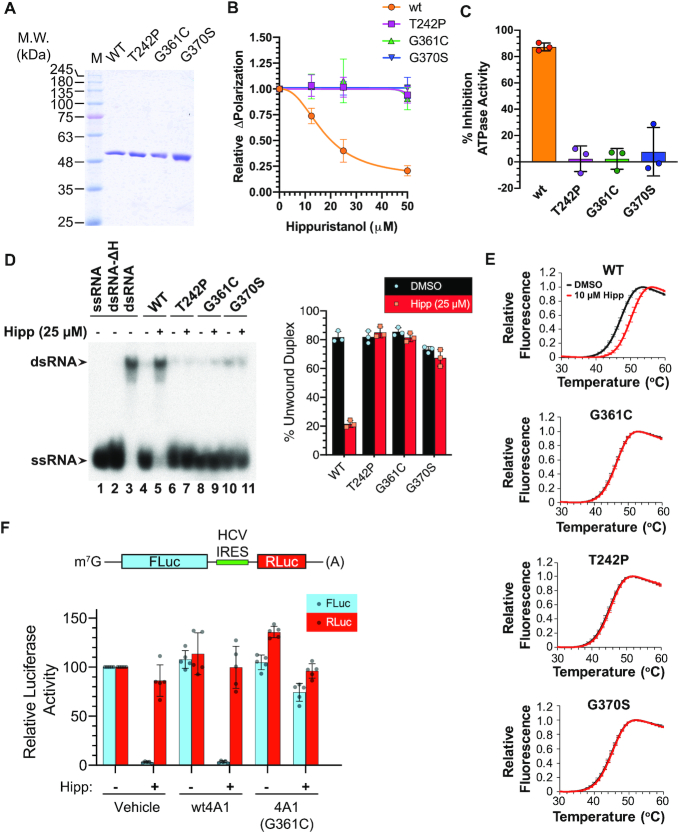
*In vitro* characterization of *EIF4A1* Hipp^R^ mutations. (**A**) Coomassie-stained 10% polyacrylamide gel of purified recombinant eIF4A1 proteins. (**B**) RNA-binding activity of *EIF4A1* allelic variants. Poly r(AG)_8_ RNA was incubated in the presence of 1 μM recombinant protein and either vehicle (0.1% DMSO) or Hipp for 30 min prior to measurements. The change in FP in the presence of Hipp relative to vehicle control is presented. *n* = 3 ± SD. (**C**) Response of ATPase activity of wt and Hipp^R^*EIF4A1* variants to 50 μM Hipp. *n* = 3 ± SD. (**D**) Helicase activity of *EIF4A1* Hipp^R^ variants in the presence or absence of Hipp. *Left panel:* The position of migration of the double-stranded (ds) RNA duplex and single-stranded (ss) RNA molecules is indicated by arrows to the left of the panel. dsRNA-ΔH: migration of radiolabelled RNA following heat denaturation of dsRNA substrate at 95°C for 5 min. *Right panel:* Quantification of three experiments is provided to the right. *n* = 3 ± SD. (**E**) DSF analysis of *EIF4A1* allelic variants in the presence or absence of Hipp. *n* = 3 ± SD. (**F**) Rescue of Hipp-mediated inhibition of translation by eIF4A1(G361C). *Top panel:* Schematic of bicistronic mRNA used in these studies. *Bottom panel:* Recombinant protein (850 ng) was added to RRL translation reactions (10 μl) containing vehicle (0.5% DMSO) or 5 μM Hipp. Reactions were incubated at 30°C for 60 min, after which firefly (FLuc) and renilla (RLuc) luciferase readings were taken. *n* = 3 ± SD.

The three mutations were re-engineered into the eIF4A1 cDNA and the proteins purified following expression in *Escherichia coli* (Figure 2A). The three mutants show similar activity to wt protein when assessed for RNA binding by fluorescence polarization assay (16) and for ATPase activity (Supplementary Figure S3). When assessed for Hipp-responsiveness, we found that only the RNA-binding activity of wt eIF4A1, but none of the Hipp^R^ variants, was inhibited by Hipp (Figure 2B). Moreover, the ATPase and helicase activities of only wt eIF4A1 were suppressed by Hipp (Figure 2C, D). Differential scanning fluorimetry (DSF) was used to probe for the recombinant proteins’ respective thermostabilities upon RNA binding and revealed that Hipp could interact with wt eIF4A1, but not with any of the three tested mutants (Figure 2E). Furthermore, experiments in which recombinant protein was added to *in vitro* translation extracts containing Hipp revealed that wt eIF4A1 was unable to rescue the inhibition, whereas the eIF4A1 (G361C) variant could (Figure 2F). *In silico* modelling of Hipp into its putative binding site within the CTD revealed that residues G370 and G361 are at the two ends of a loop that engages in extensive contacts with Hipp, while T242 is located on an adjacent loop that spans the NTD and CTD and may influence the conformation of the G361–G370 loop (Supplementary Figure S4, depicted in orange). The mechanism by which these three amino acids function to confer Hipp-resistance remains to be more directly assessed by detailed structure–activity relationship studies (see Discussion).

### Characterization of Hipp^R^ cells

**Figure 3.**
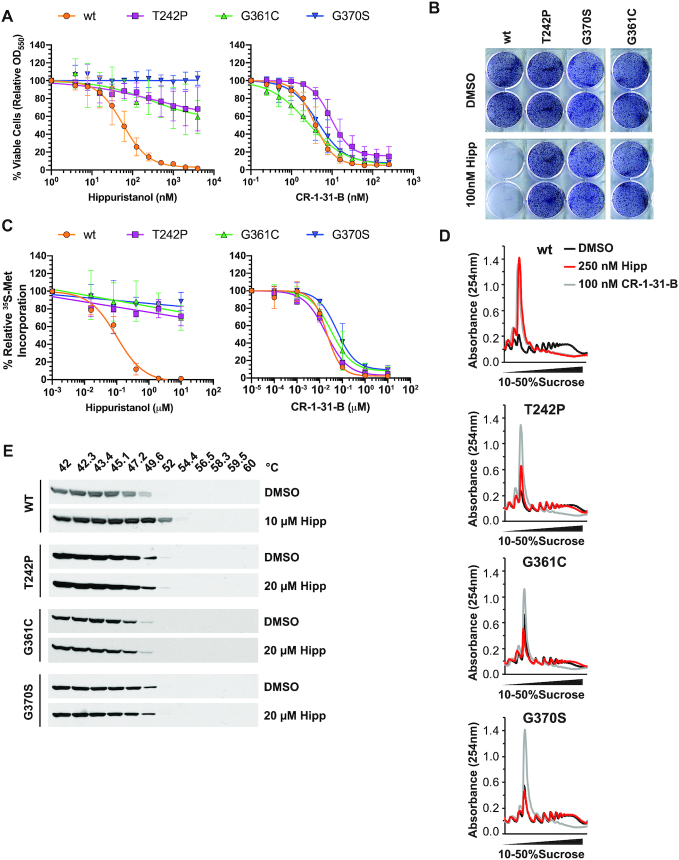
Functional characterization of Hap1 Hipp^R^ variants. (**A**) Cell viability of Hipp^R^ Hap1 cells. Cells were incubated in the presence of the indicated compound concentrations for 48 h, after which viability was assessed by SRB staining. *n* = 3 ± SD. (**B**) Crystal violet staining of the indicated Hap1 cell lines exposed to 0.1% DMSO or 100 nM Hipp for 6 days. (**C**) Assessment of protein synthesis in Hipp^R^ variants. ^35^S-Met incorporation into protein was monitored at the indicated compound concentrations. Values are expressed relative to vehicle (DMSO) controls. *n* = 3 ± SD. (**D**) Polysome profiles from the indicated cells following a 1 h exposure to CR-1–31-B or Hipp. Note there is slight variation in the height of the monosome peak between cell lines that probably reflects differences in sample loading amounts. (**E**) CETSAs performed with wt or Hipp^R^ Hap1 cells in the presence of vehicle (0.5% DMSO) or Hipp.

Exposure of all three Hipp^R^ Hap1 clones to Hipp concentrations cytotoxic to wt cells for a short- (48 h) or long- (6 days) period of time, further substantiated the Hipp-resistant phenotype of these three variants (Figure 3A, B). In contrast, all cells were sensitive to the cytotoxic effects of a second, unrelated eIF4A inhibitor: the rocaglate, CR-1-31-B (Figure 3A). Protein synthesis in all three Hipp^R^ variants was unaffected by concentrations of Hipp as high as 10 μM, as monitored by relative [^35^S]-methionine incorporation, whereas translation in wt Hap1 cells was beginning to be affected by concentrations of Hipp >0.1 μM following a 1 h exposure to compound (Figure 3C). The translational response of all cell lines to CR-1-31-B was similarly inhibited, regardless of eIF4A1 mutational status (Figure 3C). These results were also reflected by polysome profiles obtained from cells exposed to Hipp or CR-1-31-B (Figure 3D). To monitor eIF4A1 engagement by Hipp *in cellula*, we performed a cellular thermal shift assay (CETSA) (38). An increase in eIF4A1 thermostability in the presence of Hipp was seen only with wt Hap1 cells, whereas the eIF4A1 variants from the Hipp^R^ cells did not exhibit a shift in melting temperature between vehicle- and Hipp-treated cells (Figure 3E). Taken together, these results indicate that none of the eIF4A1 variants identified in our CRISPR/Cas9 screen are affected by Hipp *in cellula* under the tested conditions.

**Figure 4.**
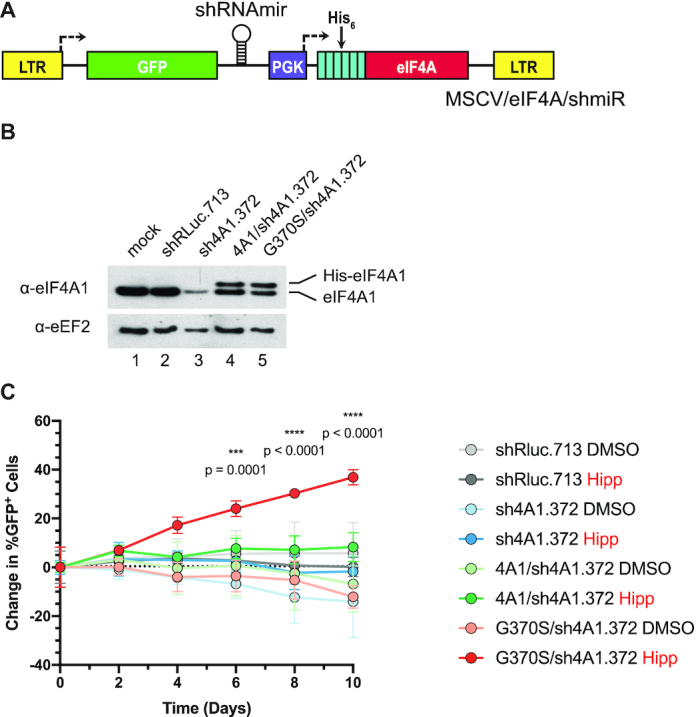
Ectopic expression of the *EIF4A1* Hipp^G370S^ allele confers Hipp resistance in NIH/3T3 cells. (**A**) Schematic diagram of MSCV co-expressing His_6_-eIF4A1 and sheIF4A1 (sh4A1). (**B**) Western blot analysis of eIF4A1 in the indicated transduced cell lines. Extracts were fractionated on a 4–12% gradient NuPAGE Bis-Tris gel, with electrophoresis performed in MES SDS running buffer as recommended by the manufacturer (Invitrogen). (**C**) Competition assay of transduced NIH/3T3 cells assessing ability of *EIF4A1*^G370S^ cDNA to confer Hipp-resistance. Transduced cells (GFP^+^) were mixed with parental cells (GFP^−^) and cultured in the presence of 250 nM Hipp for the indicated periods of time. *P*-values were calculated using the fold changes and a two-way ANOVA followed by Tukey's multiple comparison test. *n* = 3 ± SD.

We then sought to demonstrate that the mutant *EIF4A1* alleles were sufficient to confer resistance to Hipp. To this end, we took advantage of a previously employed retroviral complementation vector (RCV) system in which a miR30-based shRNA targeting *EIF4A1* is co-expressed with an shRNA-resistant His_6_-tagged *EIF4A1* cDNA (39) (Figure 4A). We engineered the wt *EIF4A1* or *EIF4A1^G370S^* allele in combination with sh4A1.372, an shRNA previously shown to knock down *EIF4A1* (40). eIF4A1 was suppressed in NIH/3T3 cells infected with MSCV/sh4A1.372, MSCV/(4A1/sh4A1.372), or MSCV/(G370S/sh4A1.372) (Figure 4B, compare lanes 3–5 to 1 and 2). Recombinant His_6_-eIF4A1 or His_6_-eIF4A1^G370S^ was present in MSCV/(4A1/sh4A1.372) and MSCV/(G370S/sh4A1.372) infected cells, respectively (compare lanes 4 and 5 to 1). Infected cells (GFP^+^) were then mixed in a 1:1 ratio with non-infected (GFP^−^) cells, followed by addition of DMSO or Hipp to the culture media.

In the presence of DMSO, there was no significant change in the proportion of GFP^+^/GFP^−^ cells over the course of 10 days for any cell line. Exposure of MSCV/shRluc.713, MSCV/sh4A1.372, MSCV/(4A1/sh4A1.372), or uninfected cells to Hipp reduced cell viability of GFP^+^ and GFP^−^ cells over the course of 10 days, but the GFP^+^/GFP^−^ ratio did not change significantly. Enrichment of GFP^+^ cells in the presence of Hipp was only seen for MSCV/(G370S/sh4A1.372)-infected cells, where levels increased 40% from a starting point of ∼50%, reaching ∼90% by the end of the experiment (Figure 4C). These results demonstrated that ectopic expression of eIF4A1^G370S^ is sufficient to confer Hipp resistance.

### Identification of Hipp-responsive mRNAs

**Figure 5.**
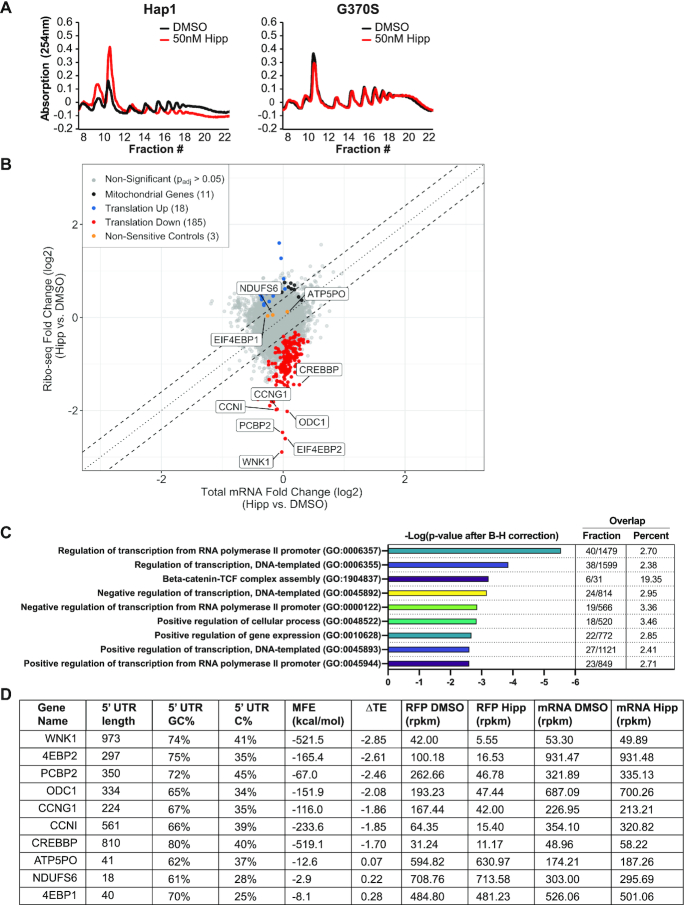
Ribosome profiling and characterization of Hipp-sensitive transcripts. (**A**) Response of cellular polysomes to transient Hipp exposure. The indicated cells were exposed to 50 nM Hipp for 1 h, after which polysomes were fractionated. (**B**) Scatter plot showing Log_2_ changes in RFP versus RNA-Seq densities (measured in RPKM) in Hipp- versus DMSO-treated cells. Hipp-sensitive genes with *P*_adj_-values < 0.05 and a log_2_ fold change < 0 are indicated in red. The genes with a *P*_adj_-values < 0.05 and a log_2_ fold change > 0 are shown in blue, while three un-responsive genes used as negative controls in subsequent experiments are shown in orange. Mitochondrial mRNAs are shown as black dots. (**C**) GO analysis predictions of the effects of Hipp on Hap1 cells. Shown are the negative logarithms of the Benjamini–Hochberg (B–H)-corrected *P*-values. Overlap represents the number of genes per GO term, represented as fractions and percentages. (**D**) Summary table of the characteristics of the seven most Hipp-sensitive genes along with three negative controls (*ATP5PO, NDUFS6* and *EIF4EBP1*).

As a selective inhibitor of eIF4A RNA binding, Hipp offers the unique opportunity to define mRNAs whose translation is most responsive to eIF4A perturbation. Titration of Hipp onto wt Hap1 and *EIF4A1*^G370S^ cells showed that a 1 h exposure to 50 nM compound inhibited protein synthesis by ∼25–50% in wt, but not *EIF4A1*^G370S^, Hap1 cells (Figures 3C and 5A). This concentration was chosen to identify those mRNAs whose recruitment into ribosomes is most sensitive to eIF4A inhibition.

We therefore defined the Hipp-responsive translatome in Hap1 cells by using ribosome profiling. Prepared libraries were highly reproducible, indicated by the strong correlation among replicates for both RNA-seq and Ribo-seq libraries (Supplementary Figure S5a). The Ribo-Seq read distribution was from 25–40 nucleotides (nts) (Supplementary Figure S5b), and an average of 15 million Ribo-Seq reads and 6 million RNA-Seq reads were found to uniquely map to a total of 16 072 annotated mRNA transcripts. The vast majority of Ribo-Seq reads were mapped to the coding sequences (CDS) with a negligible presence in 3′UTRs as compared to RNA-seq libraries (Supplementary Figure S5c, d). DESeq2 was used to determine changes in mRNA abundance and translation efficiency (TE) (30). We found no genes with significant changes (defined as having an adjusted *P*-value (*P*_adj_) < 0.05) at the level of mRNA abundance (RNA-Seq reads only). Our data showed poor to no correlation with previously published Ribo-seq sets that have examined the impact of other translation initiation inhibitors having different mechanisms of action (e.g. rocaglates [RocA, silvestrol] and mTOR inhibitors [PP242, INK128]), or those that used different concentrations of Hipp on other cell types (Supplementary Figure S6a) (see Discussion). We found a total of 203 genes to have significant changes (18 elevated and 185 decreased) in TE (Ribo-Seq reads normalized over RNA-Seq reads) as a result of Hipp treatment (Figure 5B and Supplementary Table S3). Gene ontology (GO) analysis of Hipp-sensitive mRNAs revealed that a large proportion are implicated in transcriptional regulation (*P*_adj_ = 2.872 × 10^−6^; Figure 5C). To validate these results, we chose the top seven most Hipp-responsive mRNAs (*WNK1, EIF4EBP2 [4EBP2], PCBP2, ODC1, CCNG1, CCNI* and *CREBBP*), as well as three control mRNAs (*ATP5PO, NDUFS6* and *EIF4EBP1 [4EBP1]*) (Figure 5B, D and Supplementary Table S3).

**Figure 6.**
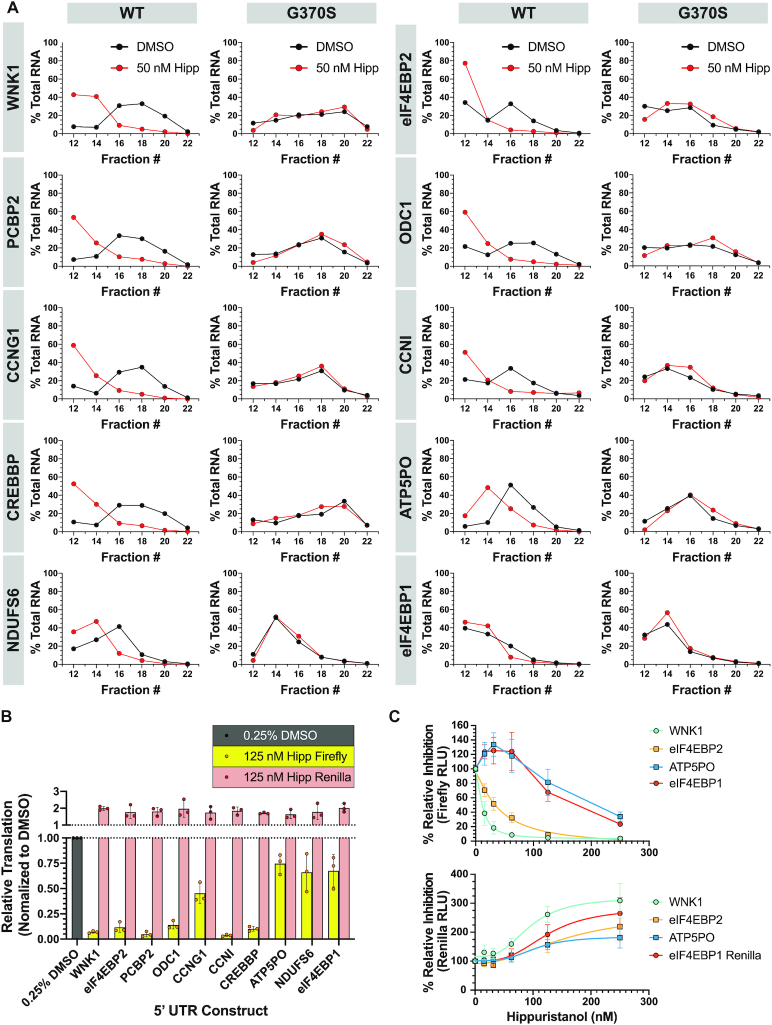
Validation of Hipp-responsive transcripts in Hap1 cells. (**A**) Polysome analysis of select Hipp -sensitive transcripts. Polysome fractions (#12–22, every 2^nd^ fraction analyzed) from 50 nM Hipp- or DMSO-treated Hap1 or G370S cells were probed for the abundance of the indicated mRNAs. Data are presented as the mean values of two biological replicates. Note that fractions at the top of the gradient (#1–11) which include free RNA and ribosomes were not included in the analysis since the RNA in these tend to be degraded. (**B**) Luciferase expression data from reporters harboring the 5′ leader region of select Hipp-responsive mRNAs. HEK-293T/17 cells were transfected with m^7^G-capped bicistronic mRNA, then 1 h later exposed to 125 nM Hipp for 7 h, at which point cells were harvested and luciferase values measured. *n* = 3 ± SD. (**C**) *In cellula* dose-response of the indicated reporters to Hipp. *n* = 3 ± SD.

We assessed the distribution of these mRNAs across polysome fractions of Hipp-treated wt Hap1 and *EIF4A1*^G370S^ cells by RT-qPCR (Figure 6A). The distribution of the seven most Hipp-responsive mRNAs showed greater shifts in polysomes from Hipp-treated wt Hap1, but not *EIF4A1*^G370S^ cells (Figure 6A). In contrast, the distribution of the control *ATP5PO, NDUSF6*, and *4EBP1* mRNAs were only slightly affected in polysomes of Hipp-treated Hap1 cells (Figure 6A). To confirm that Hipp-responsiveness was mediated through the mRNA 5′ leader region, we cloned the 5′ leader regions from the top seven hits and three control mRNAs and placed these sequences upstream of the firefly luciferase (FLuc) cistron of a bicistronic dual luciferase reporter (Figure 2F and Supplementary Table S4). Transfection of these mRNAs into HEK-293T/17 cells, followed by exposure to 125 nM Hipp for 7 h demonstrated that the 5′ leader regions of the Hipp-responsive mRNAs (*WNK1, eIF4EBP2, PCBP2, ODC1, CCNG1, CCNNI, CREBBP*) were sufficient to mediate sensitivity to Hipp, compared to the three control transcripts (*ATP5PO, NDUFS6*, and *4EBP1*) (Figure 6B). A dose titration of Hipp onto cells transfected with two of the more responsive mRNAs, *WNK1*-FF/HCV/Ren and *4EBP2*-FF/HCV/Ren, confirmed these results over a larger concentration range (Figure 6C). Here, expression from reporters harboring the *WNK1* and *4EBP2* 5′ leaders were much more sensitive to Hipp inhibition than control reporters with the *ATP5PO* or *4EBP1* 5′ leader regions (Figure 6C, top panel). This validation step provided us with the confidence required to then undertake mining of our data to identify possible correlates with Hipp-responsiveness.

**Figure 7.**
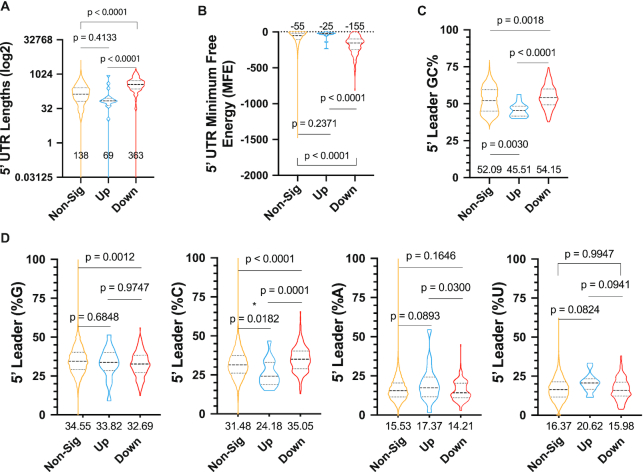
Characterization of Hipp-sensitive transcripts. (**A**) Lengths of the 5′ leader regions of each group: non-significant (*P*_adj_ ≥ 0.05, 10442 genes), up-regulated (log_2_ fold change > 0, 18 genes), and down-regulated (log_2_ fold change < 0, 185 genes) are shown with median lengths displayed. A one-way ANOVA with Tukey's multiple comparison test was used to calculate the *P*-values shown. (**B**) The non-normalized minimum free energy (MFE) of the 5′ UTRs of each group is shown along with the median values and *P*-values from a one-way ANOVA with Tukey's multiple comparison test. (**C**) The %GC composition of the 5′ leader region of each group with median values shown beneath each plot. A one-way ANOVA with Tukey's multiple comparison test was used to calculate *P*-values. (**D**) Nucleoside base compositions of the 5′ leader regions of each group are represented as percentages. Median values are displayed beneath each data set. Tukey's multiple comparison test was used to calculate *P*-values.

On a global scale, for those mRNAs whose translation was inhibited, we noted a strong correlation between Hipp-responsiveness and median 5′ leader length (138 nts in unresponsive versus 363 nts in responsive, *P* < 0.0001) (Figure 7A). Hipp-responsive transcripts also harbored more structured 5′ leaders with lower total minimum free energy (MFE) values, as calculated by RNAfold (41) (Figure 7B). When the MFE was normalized to length, there was no significant difference between Hipp-responsive and unresponsive transcripts (Supplementary Figure S7a), nor between local secondary structure (within a sliding window of 20 nts) (Supplementary Figure S7b). The %GC content was greater within the 5′ leaders of Hipp-sensitive compared to unresponsive mRNAs (median 54.15% versus 52.05%, *P* = 0.0018) (Figure 7C). The distribution of GC content along the 5′ leader region was not skewed between Hipp-responsive and -unresponsive mRNAs (Supplementary Figure S7c), but there was a slight polypyrimidine bias among Hipp-sensitive genes (Supplementary Figure S7d). The increased GC content in the Hipp-responsive transcripts was associated with a higher proportion of cytosines in their 5′ leader regions than their unresponsive counterparts (median 35.05% versus 31.48%) and a lower G content (median 32.69% versus 34.55%) (Figure 7D). Lastly, as studies linking eIF4A-dependency of mRNAs to the presence of G-quadruplexes have been reported (42), we found a difference in 5′ leader predicted G-quadruplex content between the Hipp-responsive and a randomly selected group of mRNAs (Supplementary Figure S7e). Taken together, these data indicate that increased 5′ leader length or overall secondary structure, and elevated cytosine content may be contributors to Hipp sensitivity.

**Figure 8.**
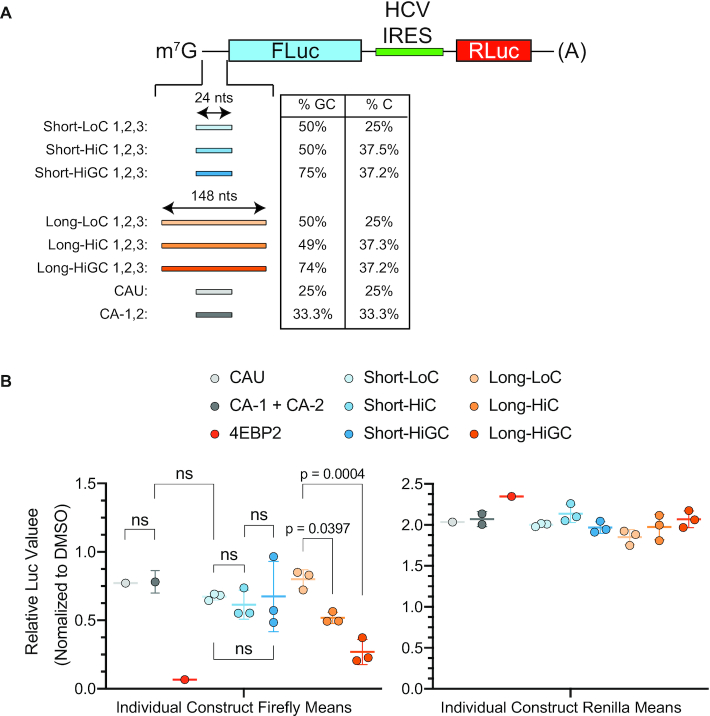
Effect of Length and Nucleotide Composition on Hipp-responsiveness in Hap1 cells. (**A**) Schematic of bicistronic mRNA used in these studies with the indicated inserts and parameters (%GC and %C content) associated with these 5′ leader regions. See Supplementary Table S5 for sequence information. (**B**) Firefly and Renilla luciferase expression data from reporters harboring synthetic constructs of varying lengths and nucleotide compositions following mRNA transfection into HEK-293T/17 cells and treatment with Hipp at 125 nM for 7 h. Each point represents the mean of biological triplicates for each construct, with three different constructs (Supplementary Table S5) being tested for each category. The mean is displayed as a colored line within each group. All values were normalized to their respective DMSO controls. *n* = 3 ± SD. Raw counts are available in Supplementary Table S6. An ordinary one-way ANOVA with Sidak's multiple comparison post-hoc test was used to calculate *P*-values.

To experimentally test the effects of 5′ leader nucleotide content and length on Hipp-dependency, we generated 21 test reporter constructs. Namely, six categories of reporters were made, each category consisting of three different constructs (Figure 8A and Supplementary Table S5). The categories were stratified according to three features—length, %GC and %C content (Figure 8A and Supplementary Table S5). Three additional reporters (negative controls) harboring no Gs were also generated (CAU-1, CA-1, CA-2). Following *in vitro* transcription, mRNA was transfected into HEK-293T/17 cells, Hipp (125 nM) added to cells, and 7 h later luciferase values determined (Figure 8B). Under these conditions, the CAU-1, CA-1 and CA-2-containing reporters were modestly responsive (Figure 8B and Supplementary Table S6). In contrast, our positive control transcript with the *4EBP2* 5′ leader sequence (297 nts, 75% GC, 35% C, MFE: –165.4 kcal/mol) was potently inhibited. Reporters with longer 5′ leaders were found to be more responsive to Hipp, as C and GC content increased (*P*_LongLoC-vs-LongHiC_ = 0.0397, *P*_LongLoC-vs-LongHiGC_ = 0.0004, *P*_LongHiC-vs-LongHiGC_ > 0.05) compared to those with shorter 5′ leaders (all *P*-values > 0.05). Among constructs in the Long 5′ leader group, sensitivity to Hipp was highest in the HiGC group (Long-HiGC [mean 0.269 FLuc expression when treated with Hipp] > Long- HiC [0.518] > Long-LoC [0.801]). Importantly, constructs in the HiC group exhibited greater sensitivity than those in the LoC group, even though their mean MFE values, as calculated by RNAfold (41), were ∼2.3-fold lower (Supplementary Table S5). Taken together, these results indicate that 5′ leader length and cytosine content are features that contribute to Hipp-responsiveness.

## DISCUSSION

Herein, we report on a simple, yet powerful approach that allowed us to identify *EIF4A1* variants in Hap1 cells that conferred Hipp-resistance. Our screen was not exhaustive and could have been expanded by using other Cas9 proteins with different PAM specificities, thus increasing the density of targeted sites. Nonetheless, we obtained missense mutations at three sites that conferred significant resistance to the cytotoxicity and inhibition of translation exerted by Hipp (Figure 3A–D). During colony isolation and characterization, we often found independent colonies harboring the same mutation, thus attesting to the robustness of the approach (Figure 1D). Resistance correlated with reduced binding of Hipp to the mutant proteins *in vitro* (Figure 2E) and in cells (Figure 3E). We note that T242P and G361C cells appeared slightly sensitive to high concentrations of Hipp, compared to the G370S line (Figure 3A). Whether this reflects subtle differences in allele activity or mRNA levels *in cellula* remains to be investigated. Addition of eIF4A1^G361C^ to *in vitro* translation reactions rescued the inhibition by Hipp (Figure 2F)—thus linking Hipp bioactivity to eIF4A1 target engagement.

The locations of the Hipp^R^ mutations are consistent with previous NMR studies mapping Hipp to the eIF4A1 CTD (Figure 1B) (21,43). We previously generated eIF4A1 mutants with alterations in the Hipp-binding site (Figure 1B), combining ^333^ARGIDVQ^339^ to ^333^ARGID**IG**^339^ or ^333^ARGIDVQ^339^ to ^333^ARGID**IP**^339^ mutations with ^358^HRIGRGGRFGR^368^ to ^358^HRIGR**T**GRFGR^368^ alterations, which we referred to as eIF4A1^IG/T^ and eIF4A1^IP/T^, respectively (21). The RNA-stimulated ATPase, RNA-binding, and helicase activities of the eIF4A1^IG/T^ and eIF4A1^IP/T^ mutants were not impaired by Hipp, thereby confirming their resistant nature (21). When tested in RRL extracts, both mutants rescued Hipp-mediated inhibition of cap-dependent translation (21). However, ectopic expression of eIF4A1^IG/T^ and eIF4A1^IP/T^ in cells failed to confer Hipp-resistance (data not published), indicating that these were compromised for full eIF4A activity. For example, eIF4A1 has recently been implicated in limiting stress granule formation in cells by reducing RNA condensation in cells (44)—an activity that we had not assessed for eIF4A1^IG/T^ and eIF4A1^IP/T^ mutants. Although our variomics screen was not designed to generate and select for double mutants, it is nonetheless striking that two of the three mutants that we identified also mapped to the ^358^HRIGRGGRFGR^368^ region, a motif implicated in RNA binding and ATP hydrolysis (45). The advantages of the approach described herein is that obtained variants must be able to support cell growth and proliferation and fulfill all functions of eIF4A1.

These former studies also explained the selectivity of Hipp for eIF4A by noting that the Hipp-interacting amino acids are only 100% conserved in eIF4A1 and eIF4A2 but not among other DEAD-box RNA helicases (21). However, these data could not discount the possibility that other cellular targets were being engaged and mediating the Hipp response, which our current study does eliminate. Modeling of Hipp into the eIF4A1 CTD revealed a loop with two of the obtained mutations at its base and the T242 amino acid residue positioned adjacently (Supplementary Figure S4). It is conceivable that changes at these positions affect loop flexibility and thus Hipp access or retention, but a better understanding of the mechanism through which these mutations confer resistance demands more refined structural studies. We note that the T242 site has been reported to be phosphorylated (https://www.phosphosite.org/homeAction) and thus consider it a possibility that this post-translational modification might be involved in regulating gate movement and Hipp binding.

In assessing the global translational response to 50 nM Hipp (∼IC_50_), we found 203 mRNAs whose translation was sensitive specifically to eIF4A1 inhibition, an assignment that we could make through the use of *EIF4A1*^G370S^ cells in validation experiments (Figure 6A). The Hipp-responsive mRNA set we identified differed from those in datasets of previous Ribo-Seq experiments assessing translation initiation inhibitors (Supplementary Figure S6). A previous study had defined the Hipp-responsive translatome in HEK-293/17 cells using 10 nM and 1 μM Hipp (16)—concentrations that in Hap1 cells would have either not affected or globally suppressed translation, respectively, and that would likely not have identified mRNAs whose translation are most sensitive to eIF4A1 inhibition. We also found poor correlation with datasets obtained with rocaglates (Roc A and silvestrol)—an unsurprising result, given the completely different mechanism of action between rocaglates and Hipp. Rocaglates act as interfacial inhibitors and produce a gain-of-function complex, where eIF4A and eIF4F are clamped onto RNA, and these complexes act as steric barriers to scanning ribosomes, leading to a global decrease in available eIF4F levels (16,17). Rocaglates are thus not suited to define cellular mRNAs whose translation are most sensitive to eIF4A1 fluctuations. Furthermore, our data show little to no correlation with PP242 or INK128 (mTOR inhibitors) datasets, which is consistent with the notion that preventing eIF4F formation by sequestering eIF4E into eIF4E:4EBP complexes impacts the translatome in a manner distinct from perturbing eIF4A activity (46).

A recent report by Waldron *et al.* (47) assessed global changes in dimethyl sulphate (DMS) reactivity (i.e. mRNA structure) and in polysome profiling of MCF7 cells treated with 150 nM Hipp (IC_50_ for translation inhibition in that cell line). In doing so, they reported a correlation between Hipp-dependency and increased 5′ leader length and found that these tended to gain more localized structure upon eIF4A inhibition. These data nicely correlate with our finding that Hipp-responsive mRNAs in Hap1 cells have longer and more structured 5′ leaders. However, Waldron *et al.* (47) noted a preference for structural rearrangements immediately upstream of the initiation codon—a correlation that awaits validation and that we did not find among the Hipp-responsive mRNA set in Hap1 cells (Supplementary Figure S7b). Our results are also consistent with the report that mRNAs with elevated 5′ leader secondary structure are more sensitive to inhibition by a dominant-negative eIF4A mutant than mRNAs with less structural features (6).

We also identified 18 mRNAs whose translation was upregulated in the presence of Hipp (Supplementary Table S3), this upregulation is likely only relative and reflects the resistance of these mRNAs to the downregulation of translation caused by Hipp treatment. The supporting evidence for that is that two of these are mitochondrial mRNAs (MT-CO3 and MT-ND1). The translation of mitochondrial mRNAs is not expected to be affected by Hipp and indeed the levels of all 13 mitochondrial mRNAs increase upon the treatment, though only two are expressed at the level sufficient for reaching the statistical significance (Supplementary Table S3).

Free eIF4A has been ascribed an eIF4F-independent function in initiation, which is to reduce eIF3j affinity for the 43S PIC to allow mRNA accommodation into the decoding site (48). This ATP-dependent, but helicase-independent, activity of eIF4A was not addressed in our experiments since Hipp does not block ATP binding (12). Although eIF4A1 is the best-characterized helicase in the translation process, several others have been implicated in initiation with functions that appear not to be redundant with eIF4A1 (e.g. DDX3X, DHX29) or that may have target-specific activities (e.g. DHX9, DHX36) (49,50). Identifying mRNAs whose translation is responsive to inhibition of these other helicases is key to providing insight into mRNA features that dictate gene expression changes and in elucidating the functional role of other DEAD/DEAH family members in translation. The scenario we defined in mammalian cells contrasts with what has been described in yeast, wherein eIF4A is required for ribosome recruitment on all mRNAs, regardless of secondary structure (51,52). In yeast, it appears that a second helicase, Ded1, is required to resolve 5′ leader structure to stimulate scanning and to suppress initiation at near-cognate initiation codons proximal to and upstream of structural barriers (52,53). Whether this signifies mechanistic differences in the initiation pathways between mammals and yeast awaits better characterization.

A previously identified feature that correlated with Hipp-responsiveness was cytosine content of the 5′ leader (47), which we also found and herein validated—the higher the content, the greater the Hipp-response, given a 5′ leader of sufficient length (Figures 7D and 8). The prediction that cytosine content and 5′ leader length impact Hipp-responsiveness was independently tested and confirmed *in cellula* using reporter constructs (Figure 8). Hipp-responsiveness does not increase when length is increased alone; rather, greater length allows the nucleotide composition to play a more significant role. In the shorter constructs tested, increasing C and GC content did not significantly affect Hipp sensitivity—but in mRNAs with longer 5′ leaders, greater C content alone was enough to increase sensitivity, and higher GC content further augmented this effect (Figure 8). These findings might suggest that greater length allows 5′ leaders to form more complex secondary and perhaps tertiary structures (due to greater degrees of freedom) that are subject to nucleotide composition and thus a higher requirement for eIF4A unwinding, whereas shorter 5′ leaders may be constrained to forming simpler duplex structures that are less dependent on GC content (54).

We have previously found that eIF4A1 and eIF4A2 have an inherent preference for polypurine RNA sequences, with poly (rC) sequences showing the weakest binding towards both helicases in fluorescent polarization assays (55). It may be that cap-proximal poly(C) rich regions require more sampling attempts by eIF4F (which requires the eIF4A helicase) before a stable clamped complex is formed. When present within the 5′ leader, poly(C) tracks may increase the chance of eIF4A dissociating from the mRNA template, thereby augmenting the dependency of that mRNA on higher eIF4A levels. We note that a cytosine-rich motif [called cytosine-enriched regulator of translation (CERT)] has been associated with mRNAs responsive to reduced levels of eIF4E and may reflect this higher Hipp-dependency noted above (56). These hypotheses will require more thorough vetting, however, using higher resolution experiments (i.e. single molecule experiments) than those undertaken herein.

Hippuristanol has proven to be a powerful tool with which to probe for eIF4A dependencies. The complete resistance of the *EIF4A1*^G370S^ mutant to concentrations of Hipp as high as 10 μM (Figure 3A) indicates that it is unlikely that Hipp is targeting another essential DEAD-box helicase and is consistent with the conserved nature of the eIF4A1 and eIF4A2 Hipp-binding site (21). Our experiments set the stage for larger-scale profiling of eIF4A (and eIF4F)-dependencies in different biological contexts to obtain a better view of the role that these factors play in gene regulation.

## DATA AVAILABILITY

The accession number for data originating from this study is GEO: GSE151687.

## Supplementary Material

gkaa662_Supplemental_Files

## Notes

Present address: Sarah A. E. Aboushawareb, Department of Family Medicine, Faculty of Medicine, McGill University.
