# Supplementary material for: Identification and characterization of hippuristanol-resistant mutants reveals eIF4A1 dependencies within mRNA 5′ leader regions
Source: Nucleic Acids Res. 2020 Aug 7;48(17):9521–37. doi: 10.1093/nar/gkaa662 (PMC7515738; doi:10.1093/nar/gkaa662)
Supplement: gkaa662_Supplemental_Files [file gkaa662_supplemental_files.zip › SUPPLEMENTAL INFORMATION.pdf]

SUPPLEMENTAL MATERIALS AND METHODS

RT-qPCR and Sanger Sequencing. For the purpose of sequencing Hipp-resistant Hap1 cells, RNA was extracted using TRIzol (Thermo Fisher Scientific) according to the manufacturer's instructions. RNA was reverse transcribed using M-MuLV Reverse Transcriptase (NEB #M0253) and an eIF4A1 specific primer (4A1_3'UTR_Rev: 5'-TGACAAGATGTCCATCCCTT-3'), according to the manufacturer's instructions. qPCRs were performed using SsoFast Evagreen Supermix (Bio-Rad #1725201) using the CFX96 PCR system.

The N- and C-terminal portions of eIF4A1 from each individual clone were PCR-amplified using the following primer pairs, respectively: N-4A1_Fwd (5'-CTAAGGATC**ATG**TCTGCGAGCC-3') and N-4A1_Rev (5'-CATTGTGGCTGACAGCAAAA-3'); as well as C-4A1_Fwd (5'-GCTGTCAGCCACAATGCCTT-3') and C-4A1_Rev (5'-CAGAGATTGAGCCCTGGCTG-3'). PCR fragments were cloned into the *Eco*RV restriction site of pBlueScript KS(+) and characterized by Sanger sequencing.

[^{35}S]-methionine Metabolic Labelling. Wild-type Hap1 and mutant cells were exposed to the indicated concentrations of Hipp for 60 mins. Fifteen minutes before the termination of the experiment, [^{35}S]-methionine/cysteine mix (11 mCi/mL; Perkin Elmer, Waltham, MA) was added. Cells were lysed in RIPA buffer (50 mM Tris-HCl [pH 7.5], 150 mM NaCl, 1% NP-40, 0.5% sodium deoxycholate, 0.1% SDS), and lysates spotted onto 3MM Whatman paper (pre-blocked with 0.1% methionine). Following incubation in cold 10% TCA for 20 mins, filters were transferred to 5% TCA, boiled for 15 mins, washed once with 5% TCA, once with 95% ethanol, and then dried. Incorporated radioactivity was quantified by scintillation counting.

Polysome analysis and RT-qPCR analysis of mRNA. Cells (1.6 x 10^7) were seeded into a 15 cm dish the day before analysis. Cells were incubated with the indicated concentration of compound for 1 h and washed twice with ice-cold PBS supplemented with 100 μg/mL cycloheximide (CHX). Cells were scraped with 1 mL PBS containing 100 μg/mL CHX, pelleted, and lysed with hypotonic lysis buffer (5 mM Tris-HCl [pH 7.5], 2.5 mM $MgCl_2$, 1.5 mM KCl, 2 mM DTT, 1% Triton X-100, 0.5% sodium deoxycholate, 100 μg/mL CHX). The extract was loaded onto a 10-50% sucrose gradient which was centrifuged at 35,000 rpm for 2:25 hours at 4°C. The gradients were fractionated while simultaneously reading the UV_{254} absorbance.

RNA was extracted from indicated fractions using TRIzol (Thermo Fisher Scientific) according to the manufacturer's instructions. RNA was reverse transcribed using M-MuLV Reverse Transcriptase (NEB#M0253) and oligo $(dT)_{23}$ VN primers (NEB # S1327) according to the manufacturer's instructions. qPCR reactions were performed using SsoFast Evagreen Supermix (Bio-Rad # 1725201) using the CFX96 PCR system. The following primers were used: NDUSF6 fwd (5'-TTCGGTTTGTAGGTCGTCAGA-3'), NDUSF6 rev(5'-TATCACCCGAGTCTCCACCTC-3'), ATP5PO fwd (5'-TTGAAGGTCGCTATGCCACA-3'), ATP5PO rev (5'-ACAGAAGCAGCCACTTTGGG-3'), WNK1 fwd (5'-TGCCAGAAAGCCGATTACGA-3'), WNK1 rev (5'-GTGCTGTGTTGGGTCCTTCT-3'), PCBP2 fwd (5'-TGCCATTCCACAGCCAGATT-3'), PCBP2 rev (5'-TCCAAACCTGCCCAATAGCC-3'), eIF4EBP2 fwd (5'-GCCCAATATCCCAGGAGTCAC-3')
eIF4EBP2 rev (5'-TCTCGAACTGAGCATCATCCC-3'), CCNG1 fwd (5'-AGAGATCCAAGCACAGAAGTGT-3'), CCNG1 rev (5'-TGCAGTACGCCCAGAAACAA-3'), ODC1 fwd (5'-AGTTGGTTTTGCGGATTGCC-3'), ODC1 rev (5'-CACGAAGGTCTCAGGATCGG-3'), CCNI fwd (5'-GCCTGCAACCAACTTCTGC-3'), CCNI rev (5'-TGGGAGCTATCCATCTGTGC-3'), CREBBP fwd (5'-TGCCAACCCTAGAAGCACTG-

3'), CREBBP rev (5'-GCTTGATGGTGGAGAGGTCC-3'), eIF4EBP1 fwd (5'-CGGAACTCACCTGTGACCAA-3'), eIF4EBP1 rev (5'-CAAACTGTGACTCTTCACCGC-3').

Purification of recombinant eIF4A1. Recombinant His_6-eIF4A1 proteins were expressed in E. *coli* strain BL21(DE3)/pLysS. Transformed bacteria were inoculated into LB medium and grown overnight at 37°C until an OD_{600} of 0.6 was reached. The induction was performed using 1 mM IPTG at 37°C for 3 hours. After harvesting, cells were lysed by sonication and the insoluble material removed by centrifugation at 25,000 x g for 40 mins. The supernatant was applied on a Ni_{2+}-NTA agarose column (Qiagen) and eluted material was dialyzed and further purified on a Q-Sepharose fast flow matrix (GE Healthcare), as previously described (1).

Fluorescence Polarization Assays. In brief, 1 μM recombinant eIF4A1 was added to 10 nM FAM-labelled poly $r(AG)_8$ RNA in a buffer containing 14.4 mM HEPES-NaOH [pH 8], 108 mM NaCl, 1 mM $MgCl_2$, 11.9% glycerol, 0.1% DMSO, 2 mM DTT and 1.25 mM AMP-PNP in the presence or absence of Hipp in black, low-volume 384-well plates (Corning 3820). Binding reactions were allowed to equilibrate for 30 mins at room temperature away from light, prior to measuring polarization values on a Pherastar FS microplate reader (BMG Labtech).

ATPase assay. ATPase assays were performed as previously described (2). In short, 1 μM γ-$_{32}$P-ATP (10 Ci/mmol) was incubated with 2.5 μM poly(U) RNA, 1 μg of purified recombinant protein (wt or mutant) and compound (50 μM Hipp) in the final buffer conditions: 2.5 mM $MgCl_2$, 1 mM DTT, 1% glycerol, 20 mM MES-KOH [pH 6.0], 10 mM KOAc. Reactions were performed for 30 mins at 25°C and quenched by adding EDTA to a final concentration of 2.5 mM. Inorganic phosphate and γ-$_{32}$P-ATP were resolved on PEI cellulose TLC plates using 1 M LiCl/0.3 M NaH_2PO4 as running buffer. The extent of ATP hydrolysis was quantitated using a Storm 840 scanner (GE healthcare).

RNA helicase assays. Helicase activity was assessed using a [$_{32}$P]-labeled RNA probe (RNA-1/RNA-11 duplex) and eIF4A1 (0.56 μM) as previously described (3). In short, the reaction was prepared by the addition of 2 μM [$_{32}$P]-labeled duplex in 1X helicase buffer (20 mM Hepes-KOH [pH 7.5], 70 mM KCl, 2 mM DTT, 1 mM $Mg(OAc)_2$), 20 μg of acetylated BSA (Ambion), and 1 mM ATP. Reactions were incubated at 35°C for 15 mins and stopped by the addition of 1X Stop Solution (10% glycerol, 0.2% SDS, 2 mM EDTA). Reaction components were separated on a 12% polyacrylamide gel (29:1; acrylamide/bisacrylamide), dried and quantitated using the Typhoon Trio Imager (GE Healthcare). Gels were also exposed to film (Kodak X-Omat).

Competition assay. Cells (20,000) from transduced (GFP_+) and non-transduced (GFP_-) NIH/3T3 cells were mixed and cultured in 12-well-plates in the presence of 250 nM Hipp or vehicle (DMSO). Every other day, cells were trypsinized and the relative population of GFP_+ cells as well as cell concentration were determined by flow cytometry (Guava EasyCyte, Millipore). DMSO- and Hipp-treated cells were split 1:3 and 1:1.5, respectively, and re-seeded into 12-well-plates. Knockdown of endogenous eIF4A1 and expression of His-tagged wt *EIF4A1* or *EIF4A1*$_{G370S}$ mutants were confirmed by Western blot. Cells were lysed using NP-40 buffer (50 mM Tris-HCl [pH 7.5], 150 mM NaCl, 0.5% NP-40, 2 mM EDTA) and lysates were subsequently separated on 10% NuPAGE and transferred onto a PVDF membrane. Antibodies used for immuno-detection included α-eIF4A1 (abcam 31217) and α-eEF2 (CST 2332).

Homology Modeling of Human eIF4A1. A homology model was built with RosettaCM (4) based on structures of the individual N- and C-terminal domains of eIF4A1 and full-length crystal structures of eIF4A3. For eIF4A1, the PDB structures 2ZU6 and 3EIQ were used (5,6). For eIF4A3, PDB structure 2HXY was used (7). When multiple copies of the same chain were available in one structure, all of those chains were used as independent templates. The first 19 residues were excluded from the homology model. The best resulting model was optimized with the FastRelax protocol using the ligand docking Rosetta scorefunction (8).

Hippuristanol Docking into eIF4A1. An energy-minimized structure of the R-isomer of Hipp, and the relevant Rosetta energy parameter files, was generated. Using the RosettaScripts scripting interface (9), Rosetta Ligand was used to dock Hipp into the eIF4A1 homology model (10). As residues known to be involved in Hipp binding have been previously identified using NMR (11), we used the NMR data to restrain the ligand docking simulations. These included residues 335, 336, 338, 343, 344, and 369-371. In brief, ambiguous atom pair constraints between the amide proton of each residue identified to be interacting with Hipp and all of the Hipp protons were included, such that the interaction with the lowest scoring (shortest distance) Hipp proton would be included in the score. A bounded constraint function that assigned a score of 0 for atom-atom distances of less than 5 Å was used.

The following command line was used to execute the Rosetta Ligand docking simulations, using the development version of Rosetta with git revision 776f82ba69b17a6e3fcb1adda9f150e5cf70a77c:

rosetta_scripts.linuxclangrelease -database $ROSDB -in:file:s 'eif4a_ligand_hippdock.pdb hippR_0001.fa.pdb' -parser:protocol ligand_dock.xml @flagsfile_hippR

The following "flags" file was used (@flagsfile_hippR):

```
-run:preserve_header
-ex1
-ex2
-packing:no_optH false
-packing:flip_HNQ true
-mistakes:restore_pre_talaris_2013_behavior true
-in:file:extra_res_fa hippR.fa.params
```

The protocol was repeated 1000 times and the resulting structures were evaluated based on the Rosetta score, ligand binding score, the satisfaction of the NMR constraints, and other metrics. The ligand_dock.xml file is available from authors upon request.

Traffic Light Reporter Assay. In brief, 1x10$_6$ 293T/17 cells from a clone expressing the TLR reporter (12) were transfected with 3 μg of either Cas9-expressing or Cpf1-expressing construct with TLR sgRNA and the cells grown for seven days. The extent of genome editing was determined by flow cytometry on a Guava EasyCyte (Millipore), by monitoring expression of mCherry. Cells were then harvested and gDNA extracted as described above. Genomic DNA (1 μg) was used in a PCR to amplify the TLR edited region using the following primers that allow Ion Torrent adaptors insertion:
TLR-Tor-adap-For:
5'-CCATCTCATCCCTGCGTGTCTCCGACTCAGGTTCATCTGCACCACCGGCAACC-3'; and
TLR-Tor-adap-Rev:
5'-CCTCTCTATGGGCAGTCGGTGATACGCCGTAGGTCAGGGTTCACAC-3'. The resulting PCRs were sequenced using an Ion Torrent personal genome machine as recommended by the manufacturer (Life Technologies) and analyzed. After removal of wt sequences, more than 10$_5$

modified sequences were retrieved and analyzed for presence of substitution relative to out of frame modifications.

***In vitro* Translation Experiments.** *In vitro* translation of capped FF/HCV/Ren mRNA was performed in rabbit reticulocyte lysate according to the manufacturer's instruction (Promega). Extracts were programmed with 4 μg/mL of *in vitro* transcribed mRNA, 85 ng/μL of recombinant eIF4A1 wt, mutant proteins, or buffer control, and reactions were incubated for 1h at 30°C in the presence of vehicle (0.5% DMSO) or 5 μM Hipp. Reactions were stopped by placing the samples on ice and luciferase activities measured on a Berthold Lumat LB 9507 luminometer. The concentration of eIF4A in reticulocyte lysate has been reported to be 0.17 μg/μL (13), so we aimed to compensate for inhibition of endogenous eIF4A1 by Hipp through supplementation, reaching a final concentration that corresponds to 50% of total wt eIF4A levels (0.085 μg/μL).

SUPPLEMENTAL FIGURE LEGENDS

Figure S1. CRISPR/Cas9 can be harnessed to generate variants with missense mutations or in-frame indels arising during repair by NHEJ. **a.** Outline of possible repair products arising from NHEJ during repair of a Cas9-induced double-stranded DNA break. X denotes any base. The red labeled N denotes base insertions arising during NHEJ. Only a few of many possibilities are shown for illustrative purposes. **b.** Frequency of substitution mutations versus indels generated by SpCas9 and Cpf1. The sequence targeted by SpCas9 and Cpf1 sgRNAs within eGFP is shown. The red and blue arrows denote the cutting sites of SpCas9 and Cpf1, respectively. The blue and red boxes indicate the location of the Cpf1 and SpCas9 PAM motifs, respectively. Seven days following infection of a cell line stably expressing the TLR reporter with an All-in-One lentivirus expressing the sgRNA and SpCas9 or Cpf1, genomic DNA was isolated and the targeted eGFP region amplified by PCR. Products were then sequenced on an Ion Torrent personal genome machine as recommended by the manufacturer (Life Technologies). **c.** Bar graph summarizing the percentage of reads that gave rise to indels versus base substitutions (missense/nonsense mutations).

Figure S2. All-in-One LeGO-based Cpf1 editing vector.

Figure S3. RNA binding and ATPase activity of eIF4A1 wt and $Hipp_R$ mutants. **a.** Fluorescent polarization values obtained with the indicated recombinant proteins using FAM-labelled poly $r(AG)_8$ RNA. The red dotted line signifies mP value obtained in the absence of protein. n=3 ± SD. **b.** ATPase activity of wt and $Hipp_R$ eIF4A1 mutants following a 30 min incubation with γ-^{32}P-ATP as substrate.

Figure S4. Hipp docked into the eIF4A1 homology model. The pose with the best Rosetta score and ligand interaction score that also satisfied all of the NMR constraints is shown. Hipp is shown in blue and red. Residues used in the NMR constraints, which have previously been shown to have an NOE with Hipp, are shown in yellow (11). Residues that undergo a chemical shift change upon Hipp binding are shown in grey. Residues that, when mutated, lead to Hipp resistance are shown and labelled in orange.

Figure S5. Ribosome footprinting quality control analyses. **a.** Pearson correlations between replicates (R1, R2, R3) and conditions from RNA-Seq analysis and Ribo-Seq, respectively. **b.** Read length distribution of ribosome footprints determined by Ribo-Seq. **c.** Read distribution among mRNAs from Ribo-Seq and RNA-Seq, respectively. **d.** Metagene plot of ribosome footprints (read counts) in DMSO- and Hipp-treated samples with positions shown relative to the 5' start codon (left) and 3' stop codon (right).

Figure S6. Spearman correlations with other studies. Heatmap depicting Spearman correlation values for the fold changes from previous ribosome profiling experiments.

Figure S7. Bioinformatic characterization of Hipp-responsive and non-responsive transcripts. **a.** The minimum free energy (MFE) normalized to length of the 5' leaders of each group is shown along with the median values and p-values from a one-way ANOVA with Tukey's multiple comparison test. **b.** Local MFE calculated as a sliding window of 20 nts with a step size of 2 nts is shown across the 5' leader region of the non-significant and down-regulated groups. Shaded region represents 95% confidence interval bands. **c.** Local %GC calculated as a sliding window of 20 nts with a step size of 2 nts for the non-significant and down-regulated groups. Shaded regions represent 95% confidence interval band. **d.** Nucleotide composition of the 5' leader region of each group represented as purine and pyrimidine percentages with median values shown beneath each plot. Tukey's multiple

comparison test was used to calculate p-values. **e.** G-quadruplexes found per 1000 bp using G4Hunter with a window size of 25 and a threshold of 1.4 (14). As control, a randomized set of transcripts equal in number to those in the Hipp-sensitive group was used in 100 repeated iterations. Non-significant group: $n = 100 \pm SD$. Using the mean and standard deviation of the randomly selected groups, a z-score of 2.14, corresponding to a two-tailed p-value of 0.0322, was calculated for the Hipp-sensitive genes.

SUPPLEMENTAL REFERENCES

1. Cencic, R., Robert, F. and Pelletier, J. (2007) Identifying small molecule inhibitors of eukaryotic translation initiation. *Methods Enzymol*, **431**, 269-302.
2. Lorsch, J.R. and Herschlag, D. (1998) The DEAD box protein eIF4A. 1. A minimal kinetic and thermodynamic framework reveals coupled binding of RNA and nucleotide. *Biochemistry*, **37**, 2180-2193.
3. Rogers, G.W., Jr., Richter, N.J. and Merrick, W.C. (1999) Biochemical and kinetic characterization of the RNA helicase activity of eukaryotic initiation factor 4A. *J Biol Chem*, **274**, 12236-12244.
4. Song, Y., DiMaio, F., Wang, R.Y., Kim, D., Miles, C., Brunette, T., Thompson, J. and Baker, D. (2013) High-resolution comparative modeling with RosettaCM. *Structure*, **21**, 1735-1742.
5. Chang, J.H., Cho, Y.H., Sohn, S.Y., Choi, J.M., Kim, A., Kim, Y.C., Jang, S.K. and Cho, Y. (2009) Crystal structure of the eIF4A-PDCD4 complex. *Proc Natl Acad Sci U S A*, **106**, 3148-3153.
6. Loh, P.G., Yang, H.S., Walsh, M.A., Wang, Q., Wang, X., Cheng, Z., Liu, D. and Song, H. (2009) Structural basis for translational inhibition by the tumour suppressor Pdcd4. *EMBO J*, **28**, 274-285.
7. Andersen, C.B., Ballut, L., Johansen, J.S., Chamieh, H., Nielsen, K.H., Oliveira, C.L., Pedersen, J.S., Seraphin, B., Le Hir, H. and Andersen, G.R. (2006) Structure of the exon junction core complex with a trapped DEAD-box ATPase bound to RNA. *Science*, **313**, 1968-1972.
8. Nivon, L.G., Moretti, R. and Baker, D. (2013) A Pareto-optimal refinement method for protein design scaffolds. *PLoS One*, **8**, e59004.
9. Fleishman, S.J., Leaver-Fay, A., Corn, J.E., Strauch, E.M., Khare, S.D., Koga, N., Ashworth, J., Murphy, P., Richter, F., Lemmon, G. *et al.* (2011) RosettaScripts: a scripting language interface to the Rosetta macromolecular modeling suite. *PLoS One*, **6**, e20161.
10. Lemmon, G. and Meiler, J. (2012) In Baron, R. (ed.), *Computational Drug Discovery and Design. Methods in Molecular Biology*. Springer, New York, Vol. 819.
11. Lindqvist, L., Oberer, M., Reibarkh, M., Cencic, R., Bordeleau, M.E., Vogt, E., Marintchev, A., Tanaka, J., Fagotto, F., Altmann, M. *et al.* (2008) Selective pharmacological targeting of a DEAD box RNA helicase. *PLoS ONE*, **3**, e1583.
12. Certo, M.T., Ryu, B.Y., Annis, J.E., Garibov, M., Jarjour, J., Rawlings, D.J. and Scharenberg, A.M. (2011) Tracking genome engineering outcome at individual DNA breakpoints. *Nat Methods*, **8**, 671-676.
13. Pause, A., Methot, N., Svitkin, Y., Merrick, W.C. and Sonenberg, N. (1994) Dominant negative mutants of mammalian translation initiation factor eIF-4A define a critical role for eIF-4F in cap-dependent and cap-independent initiation of translation. *EMBO J*, **13**, 1205-1215.
14. Bedrat, A., Lacroix, L. and Mergny, J.L. (2016) Re-evaluation of G-quadruplex propensity with G4Hunter. *Nucleic Acids Res*, **44**, 1746-1759.

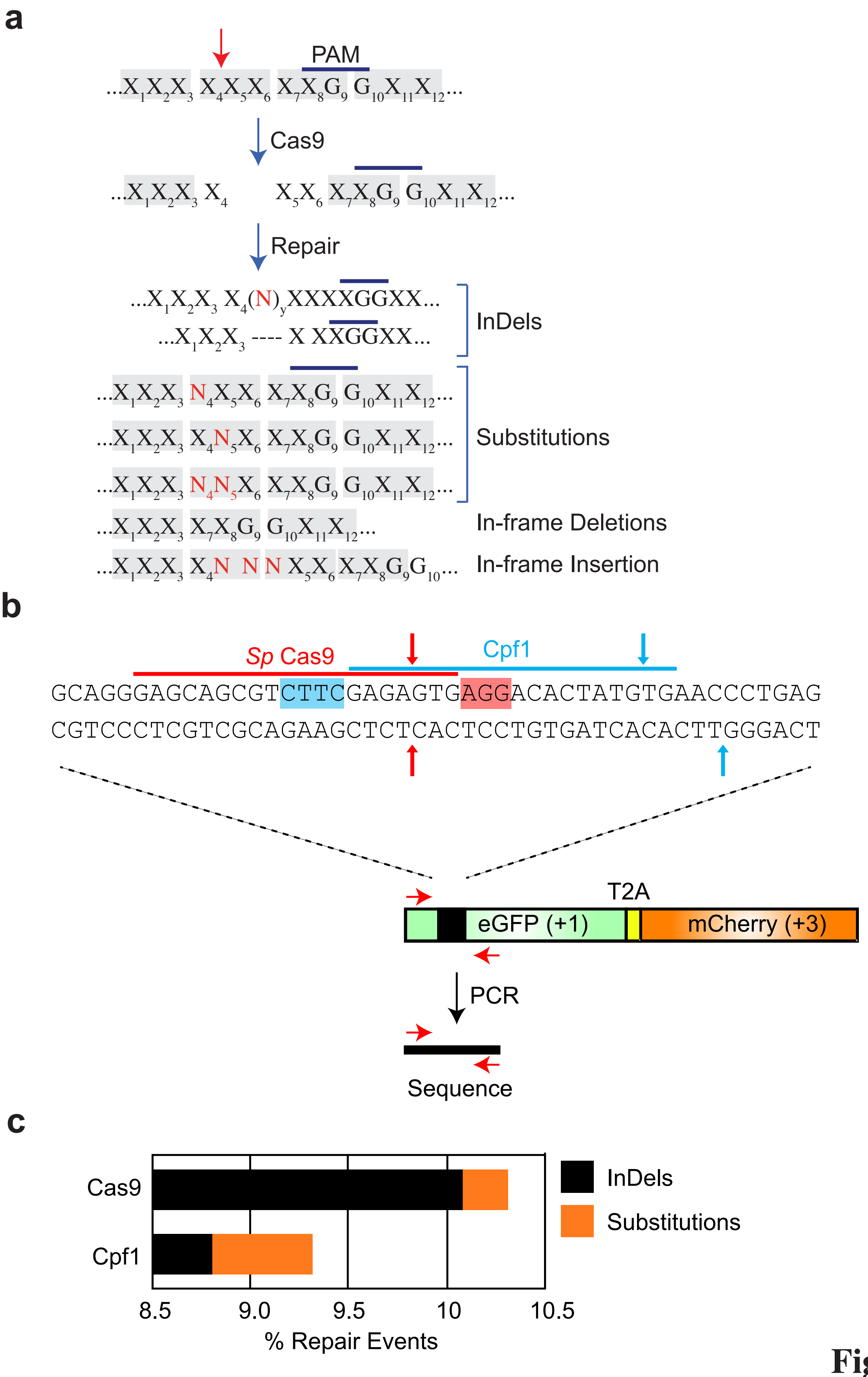
a
PAM
Cas9
Repair
InDels
Substitutions
In-frame Deletions
In-frame Insertion
b
Sp Cas9
Cpf1
GCAGGGAGCAGCGTCTTCGAGAGTGAGGACACTATGTGAACCCTGAG
CGTCCCTCGTCGCAGAAGCTCTCACTCCTGTGATCACACTTGGGACT
T2A
eGFP (+1)
mCherry (+3)
PCR
Sequence
c
Cas9
Cpf1
InDels
Substitutions
8.5
9.0
9.5
10
10.5
% Repair Events

Figure S1

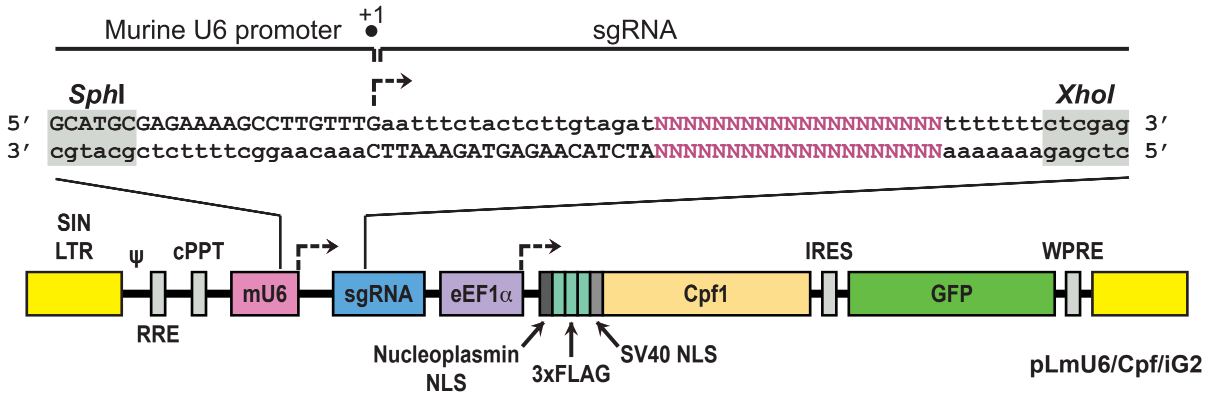

Murine U6 promoter
+1
sgRNA
SphI
XhoI
5′ GCATGCGAGAAAAGCCTTGTTTGaatttctactcttgtagatNNNNNNNNNNNNNNNNNNNNNtttttttctcgag 3′
3′ cgtacgctcttttcggaacaaaCTTAAAGATGAGAACATCTANNNNNNNNNNNNNNNNNNNNNaaaaaaagagctc 5′
SIN
LTR
ψ
cPPT
RRE
mU6
sgRNA
eEF1α
Cpf1
IRES
GFP
WPRE
Nucleoplasmin
NLS
3xFLAG
SV40 NLS
pLmU6/Cpf/iG2

Figure S2

a

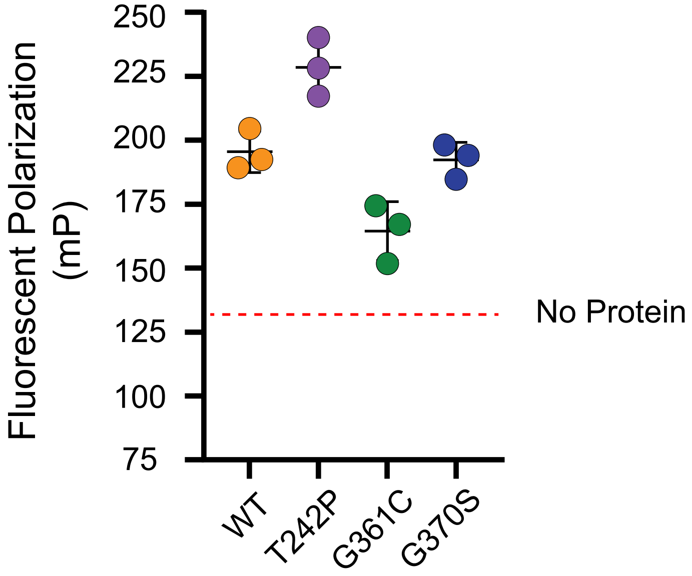
Fluorescent Polarization (mP)
250
225
200
175
150
125
100
75
No Protein
WT
T242P
G361C
G370S

b

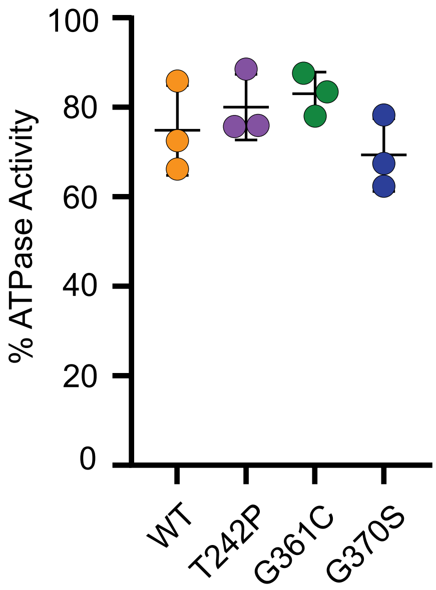
% ATPase Activity
100
80
60
40
20
0
WT
T242P
G361C
G370S

Figure S3

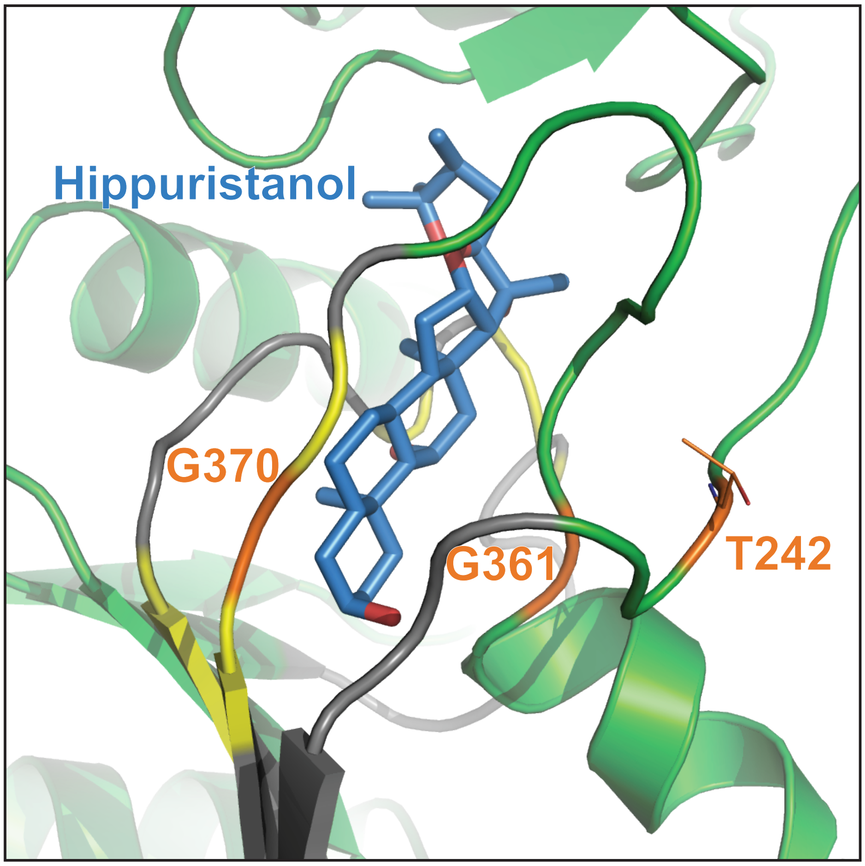

Figure S4

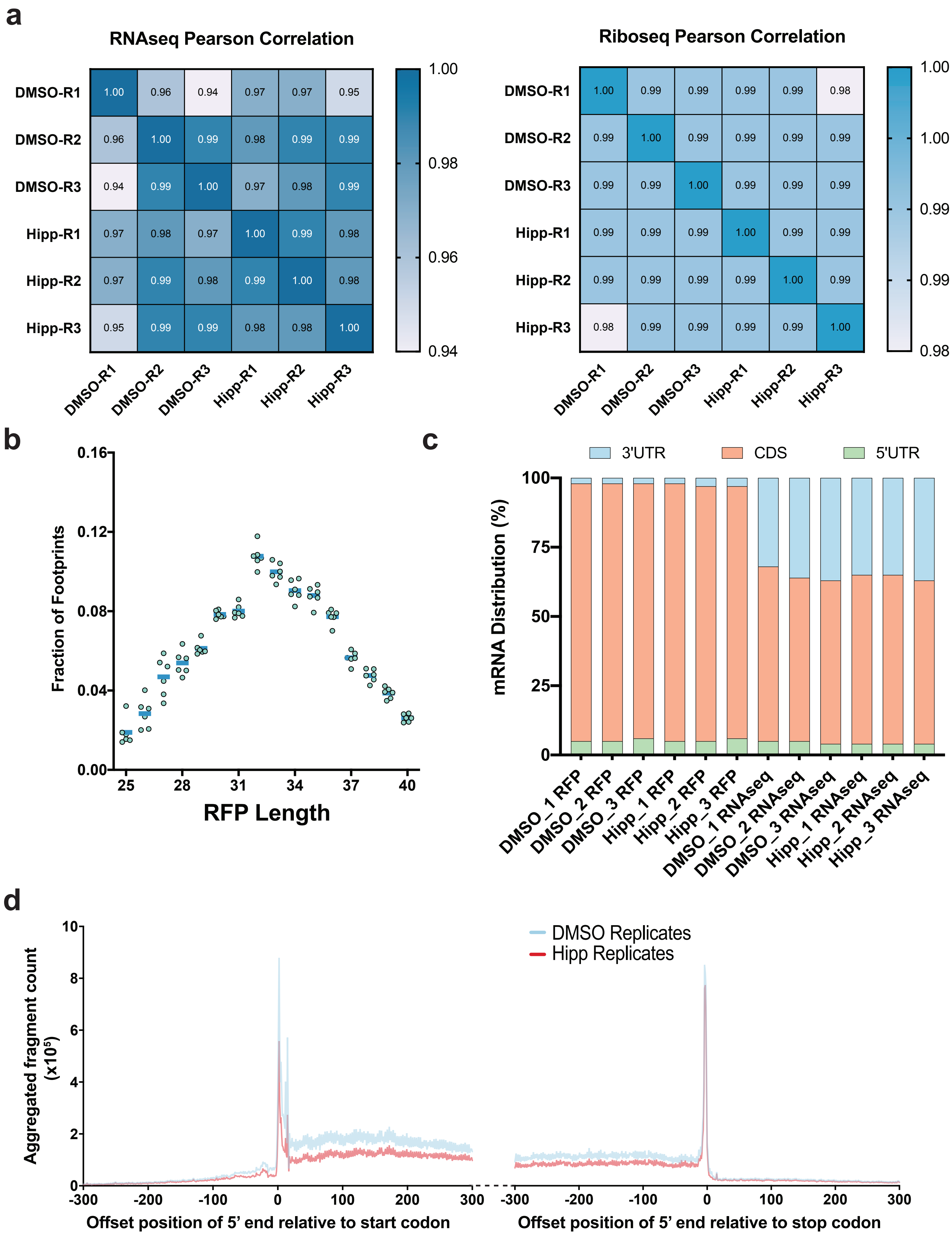

a
RNAseq Pearson Correlation
Riboseq Pearson Correlation
DMSO-R1
DMSO-R2
DMSO-R3
Hipp-R1
Hipp-R2
Hipp-R3
1.00
0.98
0.96
0.94
0.99
b
Fraction of Footprints
RFP Length
0.16
0.12
0.08
0.04
0.00
25
28
31
34
37
40
c
3'UTR
CDS
5'UTR
mRNA Distribution (%)
100
75
50
25
0
DMSO_1 RFP
DMSO_2 RFP
DMSO_3 RFP
Hipp_1 RFP
Hipp_2 RFP
Hipp_3 RFP
DMSO_1 RNAseq
DMSO_2 RNAseq
DMSO_3 RNAseq
Hipp_1 RNAseq
Hipp_2 RNAseq
Hipp_3 RNAseq
d
DMSO Replicates
Hipp Replicates
Aggregated fragment count (x10⁵)
10
8
6
4
2
0
-300
-200
-100
0
100
200
300
Offset position of 5' end relative to start codon
Offset position of 5' end relative to stop codon

Figure S5

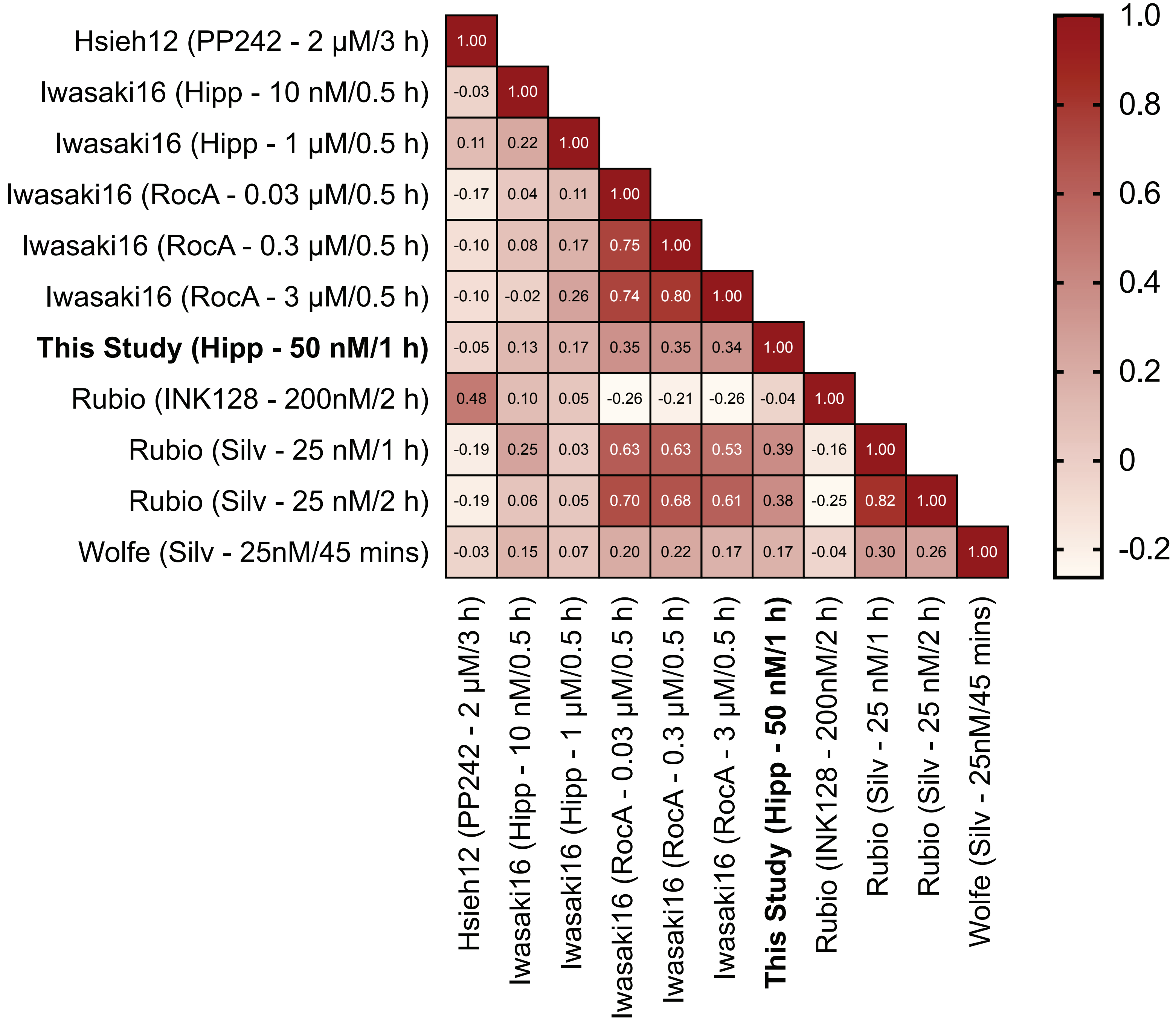
Hsieh12 (PP242 - 2 μM/3 h)
Iwasaki16 (Hipp - 10 nM/0.5 h)
Iwasaki16 (Hipp - 1 μM/0.5 h)
Iwasaki16 (RocA - 0.03 μM/0.5 h)
Iwasaki16 (RocA - 0.3 μM/0.5 h)
Iwasaki16 (RocA - 3 μM/0.5 h)
This Study (Hipp - 50 nM/1 h)
Rubio (INK128 - 200nM/2 h)
Rubio (Silv - 25 nM/1 h)
Rubio (Silv - 25 nM/2 h)
Wolfe (Silv - 25nM/45 mins)
1.00
-0.03 1.00
0.11 0.22 1.00
-0.17 0.04 0.11 1.00
-0.10 0.08 0.17 0.75 1.00
-0.10 -0.02 0.26 0.74 0.80 1.00
-0.05 0.13 0.17 0.35 0.35 0.34 1.00
0.48 0.10 0.05 -0.26 -0.21 -0.26 -0.04 1.00
-0.19 0.25 0.03 0.63 0.63 0.53 0.39 -0.16 1.00
-0.19 0.06 0.05 0.70 0.68 0.61 0.38 -0.25 0.82 1.00
-0.03 0.15 0.07 0.20 0.22 0.17 0.17 -0.04 0.30 0.26 1.00
1.0
0.8
0.6
0.4
0.2
0
-0.2

Figure S6

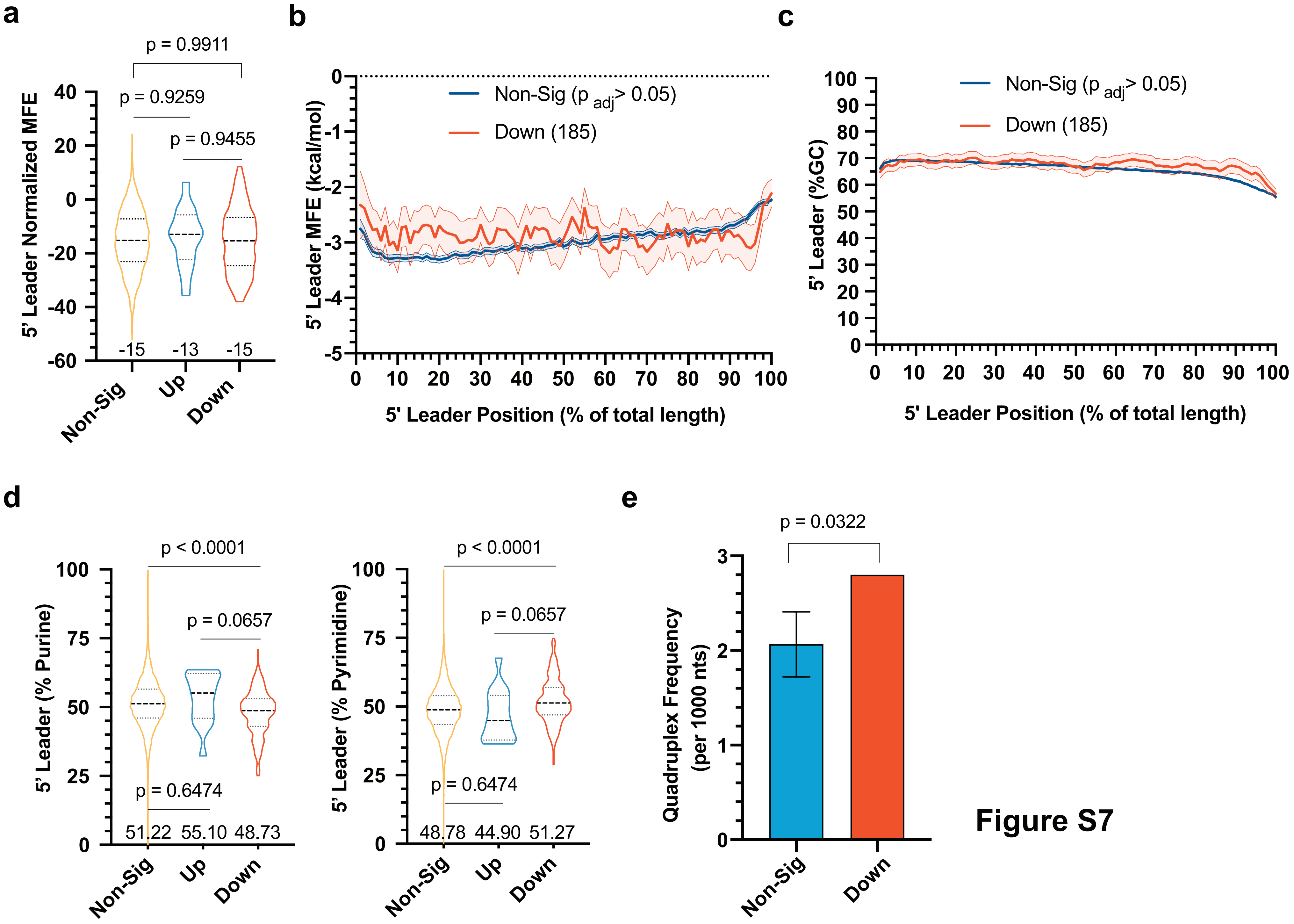
a
p = 0.9911
p = 0.9259
p = 0.9455
5' Leader Normalized MFE
-15
-13
-15
Non-Sig
Up
Down
b
5' Leader MFE (kcal/mol)
Non-Sig (p adj> 0.05)
Down (185)
5' Leader Position (% of total length)
c
5' Leader (%GC)
Non-Sig (p adj> 0.05)
Down (185)
5' Leader Position (% of total length)
d
p < 0.0001
p = 0.0657
p = 0.6474
5' Leader (% Purine)
51.22 55.10 48.73
Non-Sig
Up
Down
p < 0.0001
p = 0.0657
p = 0.6474
5' Leader (% Pyrimidine)
48.78 44.90 51.27
Non-Sig
Up
Down
e
p = 0.0322
Quadruplex Frequency (per 1000 nts)
Non-Sig
Down

Figure S7
